# A Toolkit for Targeted Neuromodulation of Striatal Direct Pathway Neurons Rescues Parkinsonian Motor Deficits in Mice

**DOI:** 10.1002/advs.202519665

**Published:** 2026-06-22

**Authors:** Zexuan Hong, Yujing Zhang, Junjiao Zhang, Hanhe Liu, Qiwei Liu, Yanglei Li, Lixin Yang, Lixia Li, Zhongjie Liu, Zhen Yuan, Zhonghua Lu, Yefei Chen, Yuantao Li, Yuwu Jiang, Taian Liu

**Affiliations:** ^1^ Department of Anesthesiology, Women and Children's Medical Center, Shenzhen Maternity and Child Healthcare Hospital Southern Medical University Shenzhen China; ^2^ Research Center for Primate Neuromodulation and Neuroimaging, Institute of Biomedical and Health Engineering, Shenzhen Institutes of Advanced Technology Chinese Academy of Sciences Shenzhen China; ^3^ Shenzhen Key Laboratory For Molecular Biology of Neural Development, Shenzhen Technological Research Center for Primate Translational Medicine, Shenzhen‐Hong Kong Institute of Brain Science, Shenzhen Institutes of Advanced Technology Chinese Academy of Sciences Shenzhen China; ^4^ Faculty of Health Sciences University of Macau Macau SAR China; ^5^ Children's Medical Center Peking University First Hospital Beijing China; ^6^ Department of Anesthesia Affiliated Shenzhen Children's Hospital of Shantou University Medical College Shenzhen Guangdong China; ^7^ Department of Neurology, The Second Affiliated Hospital and School of Brain Science and Brain Medicine Zhejiang University School of Medicine Hangzhou China; ^8^ Dreambrook Research Institute of Brain Science Hangzhou China; ^9^ Biomedical Research Institute Hubei University of Medicine Shiyan China

**Keywords:** AAV capsid, D1‐MSN, direct pathway, enhancer, parkinson's disease, selective circuit modulation

## Abstract

Striatal medium spiny neurons expressing D1 dopamine receptors (D1‐MSNs) are a key component in the direct pathway of the basal ganglia and exhibit chronically suppressed activity in Parkinson's disease. To enable selective anatomical and functional interrogation of D1‐MSNs, we developed an adeno‐associated virus (AAV) toolkit that achieved robust and selective transgene expression in D1‐MSNs through retrograde transduction of their substantia nigra axons. We first screened an AAV9 capsid insertion library and identified variants with markedly enhanced retrograde access to D1‐MSNs. Next, we engineered a series of enhancers and demonstrate that they drive strong and specific gene expression in D1‐MSNs after retrograde transduction in both mice and a macaque. Importantly, we demonstrate that our toolkit enables targeted modulation of the direct pathway, eliciting pathway‐specific behaviors and rescuing motor deficits in a murine model of Parkinson's disease. These findings highlight the utility of our D1‐MSN‐targeting tools for basic and translational research.

## Graphical abstract

An adeno‐associated virus (AAV) toolkit enables selective anatomical and functional targeting of striatal D1‐MSNs through retrograde transduction. Enhanced capsids and engineered enhancers drive robust transgene expression across murine and primate models. This system facilitates precise modulation of the direct pathway, successfully rescuing motor deficits in Parkinson's disease models and providing a valuable resource for translational research.

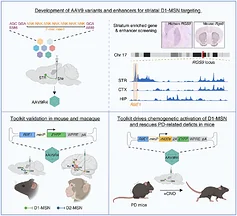

## 1 Introduction

Medium spiny neurons (MSNs), the principal output neurons of the striatum, comprise two major subtypes: D1 receptor‐expressing MSNs (D1‐MSNs) and D2 receptor‐expressing MSNs (D2‐MSNs) [1]. Both receive cortical inputs but project differently: D1‐MSNs send direct projections to the internal globus pallidus (GPi) and the substantia nigra pars reticulata (SNr), forming the direct pathway; whereas D2‐MSNs relay information to the GPi/SNr via the external globus pallidus (GPe) and then subthalamic nucleus (STN), constituting the indirect pathway. Outputs from GPi/SNr then feed back to the cortex through thalamic relays [2]. The coordinated activity of these pathways is critical for cortical information processing [3, 4]. In Parkinson's disease (PD), dopaminergic neuron degeneration in the substantia nigra pars compacta (SNc) disrupts this balance, leading to suppressed D1‐MSN activity [5, 6]. Restoring striatal D1‐MSN firing rescues bradykinesia‐like symptoms in rodent and nonhuman primate (NHP) PD models [7, 8], highlighting the therapeutic potential of targeting these MSNs.

Despite the critical role of D1‐MSNs in both basic research and therapeutic development, tools for their selective targeting and modulation remain limited. Current approaches primarily rely on Cre‐dependent transgenic mouse models [9], where optogenetic actuators can be specifically expressed in D1‐MSNs (but not D2‐MSNs) to induce functional rescue through restoration of D1‐MSN activity in mice [8]. However, these genetic methods are restricted to rodent models and lack translational applicability to higher mammals or to clinical interventions. To address this gap, researchers have developed a retrograde targeting strategy using engineered AAV capsids delivered to the SNr, which transduce D1‐MSNs via their axonal terminals [7]. Whilst effective in both rodents and NHPs, this approach has limitations: the retrograde transport motif was originally designed for cortical neurons, and the capsid itself without the MSN promoter lacks selectivity between D1‐MSNs and corticostriatal projection neurons. Additionally, while short proximal promoters enable efficient MSN expression [10], recent advances in enhancer design raise an important question [11, 12]—could more compact MSN enhancers achieve comparable expression levels whilst maintaining cellular preference? These considerations highlight the need for next‐generation D1‐MSN targeting tools optimized for both basic and translational applications.

In this study, we developed a systematic approach to identify AAV capsids and enhancers for efficient D1‐MSN targeting following SNr delivery. Our strategy involved: (1) constructing an AAV9 capsid insertion library with 7‐amino acid sequences inserted into variable region VIII (VR‐VIII) [13], and (2) implementing an RNA‐based screening protocol to enhance detection sensitivity. This approach yielded novel capsid variants demonstrating significantly enhanced transduction efficiency compared to parental AAV9 and tropism towards D1‐MSNs, but not towards cortical projection neurons. We then leveraged human ATAC‐seq data to identify four *RGS9*‐derived candidate MSN enhancers exhibiting open chromatin signatures in putamen but not cortical tissue [14, 15]. Among these, we discovered two enhancers capable of driving robust gene expression in D1‐MSNs. The resulting toolkit combines our optimized retrograde capsid with these novel enhancers, enabling both selective D1‐MSN labeling and strong transgene expression in rodent and macaque brains. Functional validation demonstrated that these tools can mediate specific designer receptors exclusively activated by designer drugs (DREADD) expression in D1‐MSNs [16], producing contraversive rotations in healthy mice and significantly improving core motor deficits in a mouse parkinsonian model.

## 2 Results

### 2.1 Selection of AAV9 Capsid Variants With Enhanced Transduction of Striatal D1‐MSNs

**FIGURE 1:**
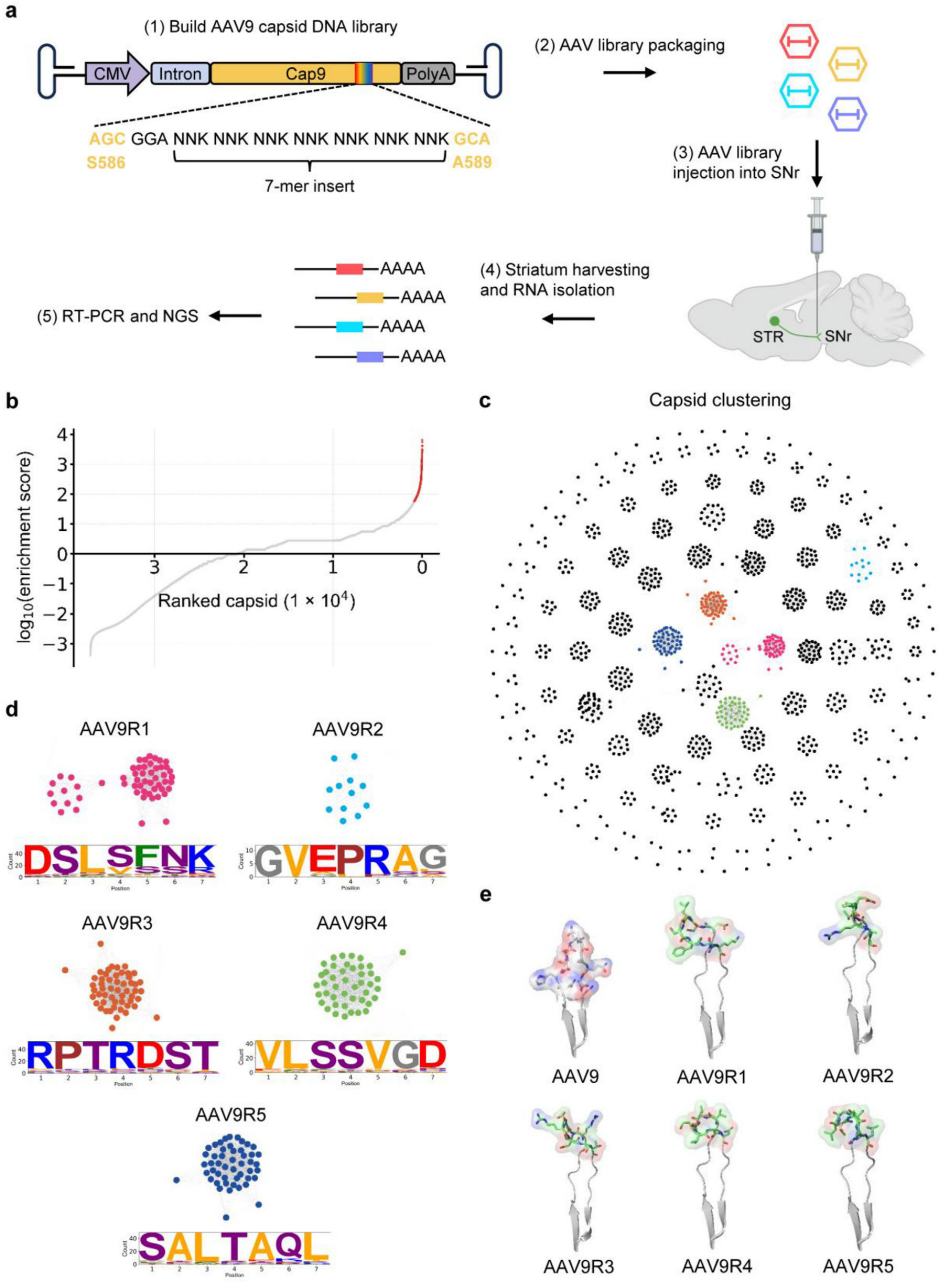
Direct evolution of an AAV9 capsid for enhanced transduction of striatonigral pathway neurons. (**a)**, Schematic showing production of the virus library and in vivo selection. SNr, substantia nigra pars reticulate; STR, striatum. (**b)**, Graph showing enrichment of capsid variants in the striatum. The enrichment score was defined as the ratio of the variant frequency in the striatal sample to that in the initial AAV pool. Variants with an enrichment score ≥ 40 are highlighted in red and subject to clustering analysis. (**c)**, Clustering analysis of enriched variants. Proximity to the center correlates with larger cluster sizes. Four large groups and one intermediate group were selected for consensus motif analysis (colored clusters). (**d)**, Consensus motifs of the five selected clusters. For each motif, the height of each amino acid logo indicates the number of variants in that cluster. (**e)**, Structural modeling of VR‐VIII regions from wild‐type AAV9 and AAV9R1∼R5 capsids using AlphaFold3.

To enable selective manipulation of the striatal direct pathway, we sought to identify AAV9 capsid variants with enhanced tropism for D1‐MSN axon terminals innervating the SNr. We employed an RNA‐based selection strategy, which captures productive transgene expression rather than mere vector genome presence—thus providing a more relevant readout of transduction efficiency. To ensure efficient expression of the capsid (*Cap*) gene in both packaging cells and mouse tissue, we replaced the native *P40* promoter with the ubiquitous *CMV* promoter (Figure 1a). Additionally, a consensus splice donor sequence was introduced to improve splicing of the cap mRNA [17].

A capsid library was generated by synthesizing a degenerate oligonucleotide containing NNK codons for seven random amino acids. This was inserted at residue 586 of the AAV9 cap gene, within the surface‐exposed variable region VIII (VR‐VIII), a site known to tolerate peptide grafting (Figure S1a, b) [18]. The original AAV9 residues at positions 587 and 588 (AQ) were replaced with glycine to alleviate conformational constraints on the downstream 7‐mer peptide insertion. The modified cap genes, flanked by AAV2 inverted terminal repeats (ITRs), were packaged into AAV particles (Figure 1a). Next‐generation sequencing (NGS) of the AAV pool revealed approximately 0.84 million unique DNA variants, with relatively even representation of all 20 amino acids, confirming high diversity and successful library construction (Figure S1c).

The viral library was bilaterally injected into the SNr of adult C57BL/6J mice at a dose of 5 × 10^8^ viral genomes (VG). Four weeks post‐injection, RNA was isolated from the striatum and reverse transcribed (Figure 1a). Sequencing of the recovered AAV cap mRNA led to the identification of over 0.14 million unique sequences. To prioritize functional variants, we calculated an enrichment score for each peptide—defined as the ratio of its frequency in the striatal sample to that in the initial AAV pool. A total of 936 peptides with enrichment scores ≥ 40 were selected for further bioinformatic analysis (Figure 1b).

We observed substantial differences in amino acid composition between the original library and the striatal‐enriched peptides (Figure S1c), suggesting selective pressure during transduction. We hypothesized that functional capsids share sequence similarity at key positions and would be recovered repeatedly. Therefore, we clustered the enriched peptides and identified consensus motifs for each group. For further characterization, we selected the four largest clusters, DSLSFNK, RPTRDST, VLSSVGD, and SALTAQL (which had 37, 53, 44 and 49 variants, respectively) and one intermediate cluster GVEPRAG with 12 variants, and designated these AAV9R1∼R5 (Figure 1c, d, and Table S1).

Using AlphaFold3 [19], we modeled the VR‐VIII structures of both wild‐type AAV9 and the peptide‐modified variants (Table S2). The inserted peptides were found to project from the apices of the surface loops, potentially reshaping the virion's surface topology and thereby altering its tropism (Figure 1e). The structural divergence among AAV9R1∼R5 variants further suggests that each peptide may impart distinct transduction properties.

### 2.2 In Vivo Characterization of AAV9R1∼R5

To evaluate whether the selected peptides enhanced the retrograde transport capacity of AAV9 capsid variants, we produced these viral variants, packaging a single stranded *hSyn* promoter‐driven *mCherry* expression cassette. For comparison, we included the parental AAV9 vector, as well as rAAV2‐retro and AAV8R12, two previously developed retrograde AAVs carrying a 10‐mer peptide insertion (LADQDYTKTA) at VR‐VIII region of AAV2 and AAV8, respectively [7, 20]. Notably, the peptides incorporated in AAV9R1∼R5 were distinct from that of rAAV2‐retro. All vectors were efficiently produced, yielding comparable titers (Table S3).

**FIGURE 2:**
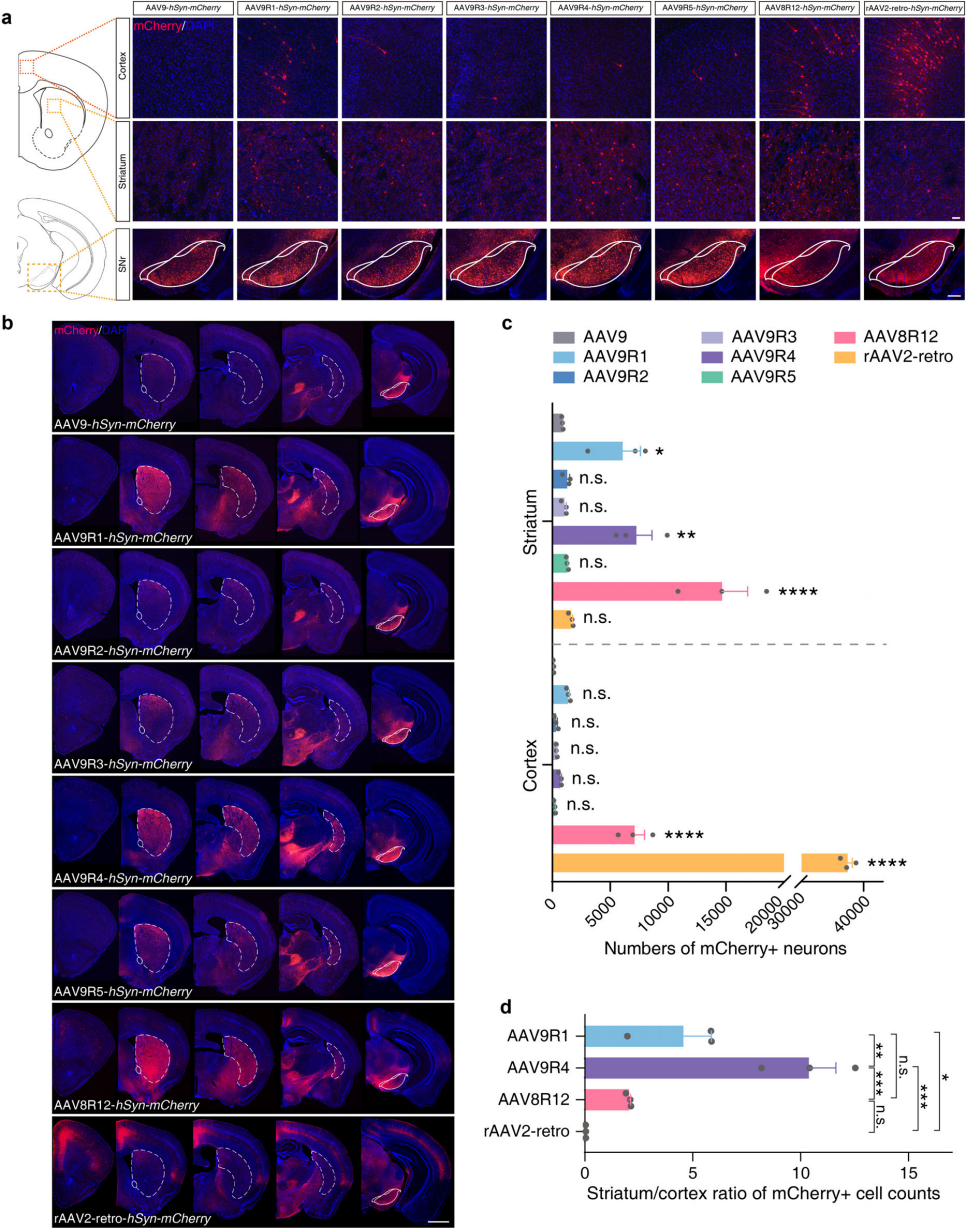
Transduction patterns of selected AAV9 capsid variants after nigral injection. (**a)**, Representative images of labeled cortex neurons, striatal MSNs and SNr following nigral injection of retrograde AAVs (rAAV2‐retro, AAV8R12, AAV9, and AAV9R1∼5). Transduction efficiency is a function of the number of mCherry+ cells, which are labeled red. Scale bars, 50 µm (top), 200 µm (bottom). (**b)**, Coronal images showing representative labeling profiles in the brain following nigral injections of retrograde AAVs (rAAV2‐retro, AAV8R12, AAV9, and AAV9R1∼5). Dashed lines mark the striatum. Solid lines mark the substantia nigra. Scale bar, 1 mm. (**c)**, Quantification of cells expressing mCherry in the striatum and cortex; n = 3 mice per group, data are represented as mean ± SEM, one‐way ANOVA with post‐hoc Tukey's test. Statistical comparisons are carried out between AAV9 and other capsid variants, *p < 0.05, **p < 0.01, ****p < 0.0001, n.s., not significant. (**d)**, The AAV9R1 and AAV9R4 variants specifically transduce the striatonigral pathway neurons, which is reflected by the striatal‐to‐cortical ratio of labeled cell density; n = 3 mice per group. AAV9 was omitted from the striatal‐to‐cortical ratio analysis to avoid artifactual inflation because of the sparse labeling in both regions. Data are represented as mean ± SEM, one‐way ANOVA with post‐hoc Tukey's test, *p < 0.05, **p < 0.01, ***p < 0.001, n.s., not significant.

Each vector was injected into the left SNr of adult mice at a dose of 2 × 10^9^ VG per animal (Figure 2a). Three weeks post‐injection, brain tissues were harvested and examined for mCherry expression in the striatum. Quantification of mCherry‐positive neurons revealed markedly enhanced retrograde transport for AAV9R1 (approximately 7.00 ± 1.02‐fold increase) and AAV9R4 (approximately 8.35 ± 0.90‐fold increase) relative to the parental AAV9 vector. However, AAV9R2, AAV9R3, and AAV9R5 showed minimal or no improvement. In addition, rAAV2‐retro exhibited retrograde labeling levels that were comparable to AAV9 (Figure 2b, c), consistent with the previously reported limited efficiency of rAAV2‐retro in transducing striatonigral pathway neurons [7]. To quantify the proportion of transduced neurons, we performed co‐immunostaining for mCherry and the neuronal marker NeuN. Both AAV9R1 and AAV9R4 demonstrated significantly higher neuronal transduction efficiencies (6.47% and 7.90%, respectively), compared to the parental AAV9 vector, which yielded only 0.60%. (Figure S2a, b).

We further analyzed mCherry expression in coronal sections across the whole brain. As expected, rAAV2‐retro effectively labeled cortico‐nigral projections, and AAV8R12 transduced both cortical and striatal neurons. In contrast, AAV9R1∼R5 displayed a more specific tropism for basal ganglia circuits (Figure 2b and Figure S2c). For example, AAV9R1 and AAV9R4 labeled many more neurons in striatum than in the cortex (Figure 2b–d), whilst AAV9R3 and AAV9R5 only promoted the transduction of neurons at the injection site, indexed by the high intensity labeling of substantia nigra and neuronal fibers in the striatum compared to the parental AAV9 (Figure 2b). We also injected AAV9R1∼R5 into other brain regions, including the medial prefrontal cortex, superior colliculus, primary motor cortex, striatum, and the dorsal lateral geniculate nucleus. In these regions, we observed that labeled cells were restricted to the areas around the injection site with scarce retrograde labeling (Figure S3). Together, these results demonstrate the effectiveness of our AAV capsid screening strategy and identified two novel AAV9 variants—AAV9R1 and AAV9R4—with enhanced retrograde transduction of striatal neurons with limited labelling in other regions projecting to SNr.

### 2.3 Identification of Striatal MSN‐Specific Gene Enhancers

**FIGURE 3:**
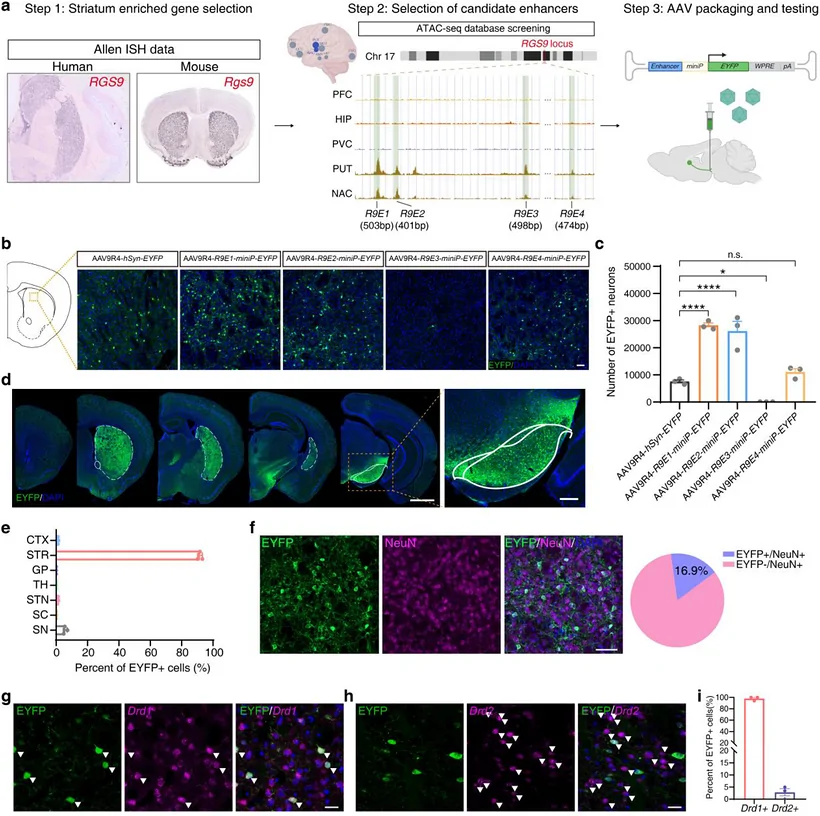
Identification and testing of robust striatal neuron enhancers. (**a)**, Pipeline for the discovery and validation of striatal enhancers. Step 1: analysis of the ISH data in the Allen Brain Atlas identified *RGS9* as a marker gene for both human and mouse striatum. Step 2: screening of a human ATAC‐seq database for open chromatin regions (OCRs). We selected OCRs of *RGS9* with specific accessibility peaks in the putamen (PUT) and nucleus accumbens (NAC) as putative striatal MSN enhancers. Step 3: four putative enhancers (*R9E1*∼*R9E4*) were packaged into AAV9R4 and screened for driving robust EYFP expression in striatum via nigral injection. (**b)**, Representative images of labeled striatal MSNs following nigral injection of AAV9R4‐*R9Ex‐miniP‐EYFP*. Transduction efficiency is a function of the number of EYFP+ cells, which are labeled green. Scale bar, 50 µm. (**c)**, Quantitation of EYFP+ cells in striatum; n = 3 mice per group, data are represented as mean ± SEM, one‐way ANOVA with post‐hoc Tukey's test, *p < 0.05, ****p < 0.0001, n.s., not significant. (**d)**, Whole‐brain images showing representative labeling profiles and higher‐magnification images of the substantia nigra following nigral injections of AAV9R4‐*R9E1‐miniP‐EYFP*. Dashed lines mark the striatum. Solid lines mark the substantia nigra. Scale bars, 1 mm (left), 200 µm (right). (**e),** Percentages of labeled neurons by nigral AAV9R4‐*R9E1‐miniP‐EYFP* injection in the different brain regions across the brain. CTX: cortex, STR: striatum, GP: globus pallidus, TH: thalamus, STN: subthalamic nucleus, SC: superior colliculus, SN: substantia nigra. n = 3 mice per group, data are represented as mean ± SEM. (**f),** Retrogradely labeled striatal neurons (green) with NeuN staining (magenta) and quantitation of EYFP+ cells amongst NeuN+ cells. n = 3 mice per group. (**g, h)**, Striatal neurons (green) retrogradely labeled by AAV9R4‐*R9E1‐miniP‐EYFP*, with *Drd1* ISH (**g**, magenta, arrowheads) or *Drd2* ISH (**h**, magenta, arrowheads). Scale bars, 20 µm. (**i)**, Quantitation of *Drd1*+ and *Drd2*+ cells amongst all EYFP+ cells; n = 3 mice per group, data are represented as mean ± SEM.

To identify enhancers specific to striatal MSNs, we first examined marker gene expression in the human striatum using the Allen Brain Atlas for guidance. The *RGS9* gene, which encodes a GTPase‐activating protein [21], was found to be selectively expressed in both human and mouse striatal regions. We then analyzed publicly‐available ATAC‐seq datasets, including the Brain Open Chromatin Atlas (BOCA) from the Roussos lab [15], and identified four striatum‐specific open chromatin regions (OCRs) within the *RGS9* locus. These candidate enhancer sequences, designated *R9E1* to *R9E4*, corresponding to regions with strong chromatin accessibility peaks (Figure 3a and Table S4). We cloned each enhancer upstream of a minimal *CMV* promoter (*CMVmini2*) and an *EYFP* reporter gene in AAV vectors.

To assess enhancer activity, the constructs were packaged into AAV9R4 and injected into the SNr (Figure 3a). Among the four candidates, we observed that *R9E1* and *R9E2* had significantly higher enhancer activity than the commonly used *hSyn* promoter did in labeling striatal neurons (approximately 3.73 ± 0.07‐fold and 3.45 ± 0.27‐fold increase, respectively), and *R9E1* had the most robust expression (Figure 3b, c). To comprehensively profile the *R9E1* enhancer activity, we packaged *R9E1*‐driven *EYFP* into AAV‐PHP.eB and delivered the virus systematically, using *hSyn*‐driven *EYFP* as a control. The results showed that the expression of EYFP promoted by *R9E1* was largely restricted to the striatum, in contrast to the widespread EYFP signal in *hSyn* group (Figure S4a). Moreover, *R9E1* displayed enhanced *Drd1*/*Drd2*+ neuron selectivity, compared with *hSyn*, in immunofluorescence analysis of several subtype neuron markers like *Drd1*, *Drd2*, parvalbumin, somatostatin, and ChAT (Figure S4b, c).

We next characterized the expression pattern after nigral delivery of AAV9R4‐*R9E1‐miniP‐EYFP*. We found that 91.7 ± 1.2% of labeled neurons were distributed in the striatum, with a corresponding neuronal transduction efficiency of 16.9% (Figure 3d–f). Then we evaluated the specificity of retrograde labeling by AAV9R4‐*R9E1‐miniP‐EYFP*. Co‐immunostaining for EYFP and either *Drd1* or *Drd2* revealed that the majority of EYFP‐positive cells were *Drd1*‐positive, with fewer than 5% co‐expressing *Drd2* (Figure 3g–i). Consistently, simultaneous nigral injection of AAV9R4‐*R9E1‐miniP‐EYFP* and striatal injection of AAV9‐*R9E1‐miniP*‐*DIO*‐*tdTomato* into Drd1‐Cre and Drd2‐Cre mice confirmed that over 98% of the MSNs labeled by our toolkit were *Drd1*‐positive. (Figure S4d–f).

These results demonstrate that the combination of the engineered AAV9R4 capsid and human‐derived *R9E1* enhancer yields a AAV toolkit for highly selective and efficient transduction of D1‐ MSNs.

### 2.4 AAV9R4 and the R9E1 Enhancer Enable Robust Retrograde Labeling of D1‐MSNs in Nonhuman Primates

**FIGURE 4:**
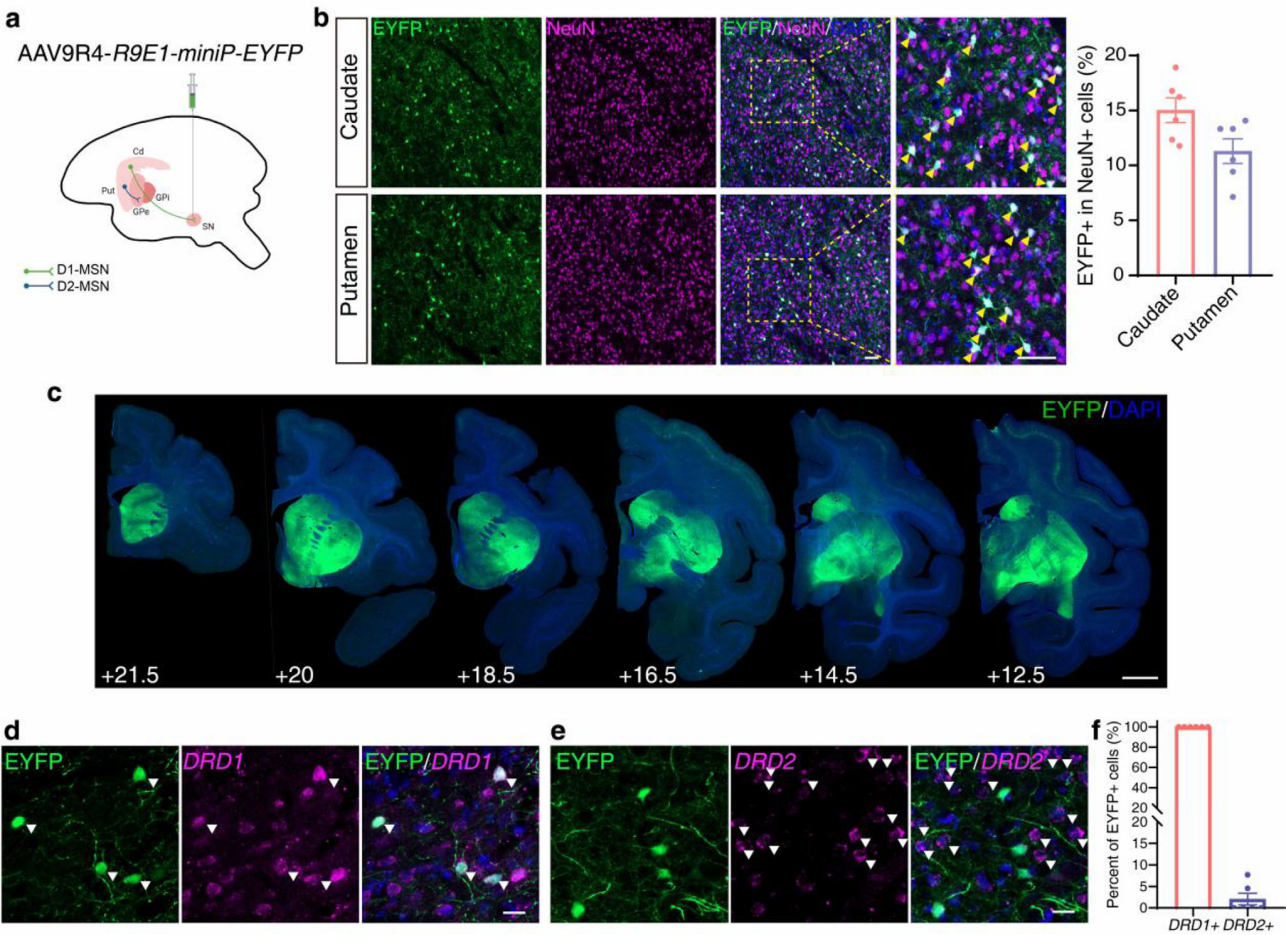
Robust labeling of D1‐MSNs after nigral injection of AAV9R4‐*R9E1‐miniP‐EYFP* in a cynomolgus macaque. (**a)**, Schematic illustrating the strategy for selective labeling of D1‐MSNs in a macaque. AAV9R4‐*R9E1‐miniP‐EYFP* was injected into the SNr of a cynomolgus macaque. Cd: caudate. Put: putamen. (**b)**, Representative high magnification images of labeled cells (green) counterstained with NeuN (magenta) and quantitation of EYFP+ cells amongst NeuN+ cells in the caudate and putamen. Arrowheads indicate EYFP cells co‐stained with NeuN. Scale bars, 100 µm. (**c)**, Representative images of retrograde labeling throughout the basal ganglia. Positions of coronal sections along the anterior‐posterior axis are indicated by the distance from EBZ (ear bar zero). Scale bar, 5 mm. (**d, e)**, Striatal neurons (green) retrogradely labeled by AAV9R4‐*R9E1‐miniP‐EYFP*, with *DRD1* ISH (**d**, magenta, arrowheads) or *DRD2* ISH (**e**, magenta, arrowheads). Scale bars, 20 µm. (**f)**, Quantitation of *DRD1*+ and *DRD2*+ cells amongst all EYFP+ cells; n = 6 sections from 1 animal, data are represented as mean ± SEM.

To enhance the translational relevance of this AAV toolkit for targeting the striatal direct pathway, we administered AAV9R4‐*R9E1‐miniP‐EYFP* into the SNr of a cynomolgus macaque (*Macaca fascicularis*) (Figure 4a). Four weeks post‐injection, strong EYFP fluorescence was observed in the caudate and the putamen, with minimal signal detected in other brain regions (Figure 4b, c). About 10%– 15% of neurons had been transduced, which is comparable to those observed in mice (Figures 3f and 4b). Analysis following immunostaining for EYFP in combination with dopamine receptor markers revealed that approximately 20% of D1‐MSNs were transduced, whereas transduction of D2‐MSNs was scarce (Figure 4d–f). These findings underscore the translational potential of our system by showing its efficacy in non‐human primates.

### 2.5 An AAV Toolkit Enables Chemogenetic Activation in the Direct Pathway of the Basal Ganglia and Elicits Robust Behavioral Phenotypes

**FIGURE 5:**
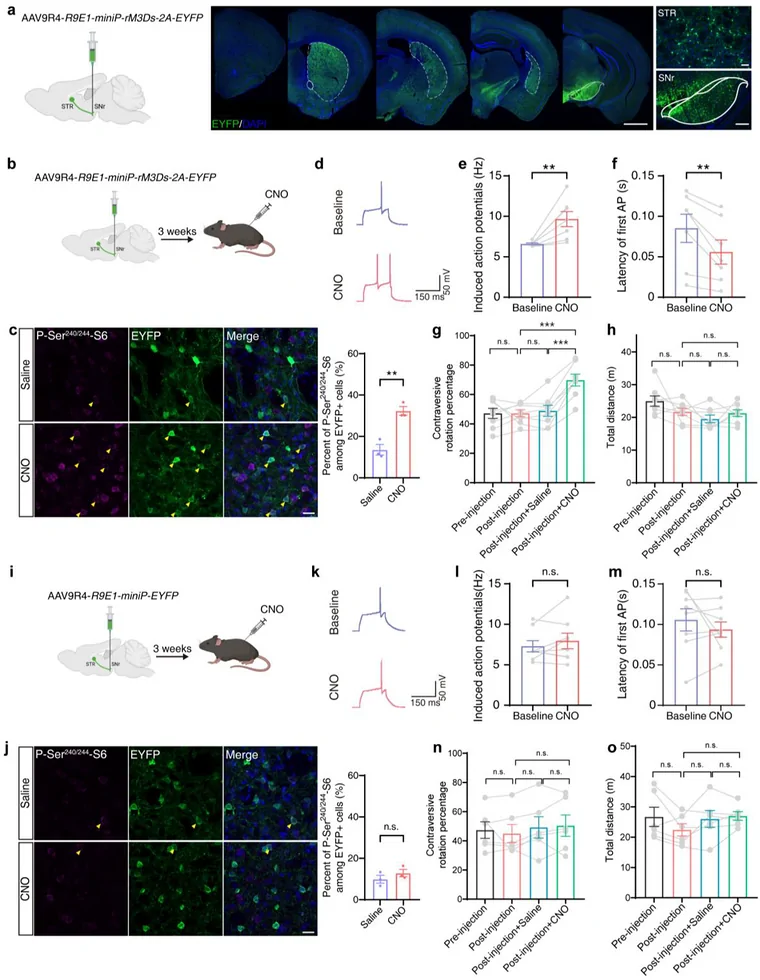
Chemogenetic activation of D1‐MSNs mediated striatal direct pathway drives robust behavior in mice. (**a)**, Left, schematic showing SNr injection of AAV9R4‐*R9E1‐miniP‐rM3Ds‐2A‐EYFP*. Middle, coronal sections of mouse brain showing labeled neurons; Right, higher‐magnification images of the striatum and substantia nigra. Dashed lines mark the striatum. Solid lines mark the substantia nigra. Scale bars, 1 mm (middle), 50 µm (upper right), 200 µm (lower right). (**b)**, Schematic showing chemogenetic activation of the striatal direct pathway following AAV9R4‐*R9E1‐miniP‐rM3Ds‐2A‐EYFP* injection and intraperitoneal injection of CNO. (**c),** Immunofluorescence for phosphorylated ribosomal protein S6 at Serine 240 and 244 residues (P‐Ser^240/244^‐S6, magenta) of labeled striatal neurons (green) and quantification of P‐Ser^240/244^‐S6+ cells among EYFP+ cells. Arrowheads indicate EYFP cells co‐stained with P‐Ser^240/244^‐S6. Scale bar, 20 µm. n = 3 mice per group, data are represented as mean ± SEM, two‐tailed unpaired *t*‐test, **p < 0.01. (**d–f),** Electrophysiological response to CNO in labeled D1‐MSNs after nigral AAV9R4‐*R9E1‐miniP‐rM3Ds‐2A‐EYFP* injection. Representative traces (**d**), quantification of action potentials (**e**), and latency of first AP (**f**) induced by depolarizing current injection at baseline and after CNO application. n = 7 cells from 6 mice, data are represented as mean ± SEM, two‐tailed paired *t*‐test, **p < 0.01. (**g**, **h),** Behavioral alterations following activation of the direct pathway in mice. Quantification of the percentage of contraversive rotation made of the total number of rotations (**g**) and the total distance (**h**) traveled in an open field test; n = 8 mice per group, data are represented as mean ± SEM, one‐way ANOVA with Tukey's post‐hoc test, ***p < 0.001, n.s., not significant. (**i)**, Schematic showing a unilateral AAV9R4‐*R9E1‐miniP‐EYFP* injection into the mouse SNr. (**j)**, Immunofluorescence for P‐Ser^240/244^‐S6 (magenta) of labeled striatal neurons (green) and quantification of P‐Ser^240/244^‐S6+ cells among EYFP+ cells in AAV9R4‐*R9E1‐miniP‐EYFP* injected mice. Arrowheads indicate EYFP cells co‐stained with P‐Ser^240/244^‐S6. Scale bar, 20 µm. n = 3 mice per group, data are represented as mean ± SEM, two‐tailed unpaired *t*‐test, n.s., not significant. (**k–m),** Electrophysiological response to CNO in labeled D1‐MSNs after nigral AAV9R4‐*R9E1‐miniP‐EYFP* injection. Representative traces (**k**), quantification of action potentials (**l**), and latency of first AP (**m**) induced by depolarizing current injection at baseline and after CNO application. n = 8 cells from 6 mice, data are represented as mean ± SEM, two‐tailed paired *t*‐test, n.s., not significant. (**n, o),** No behavioral alterations were observed in control mice. Quantification of the percentage of contraversive rotation made of the total number of rotations (**n**) and the total distance (**o**) traveled in an open‐field test; n = 6 mice per group, data are represented as mean ± SEM, one‐way ANOVA with Tukey's post‐hoc test, n.s., not significant.

The direct pathway of the basal ganglia, mediated by D1‐MSNs, plays a critical role in motor control [22]. Chemogenetic activation of unilateral D1‐MSNs reliably induces contralateral rotational behavior in mice. Using our engineered capsid and striatal enhancer, we expressed either rM3Ds or hM3Dq to evaluate their suitability for targeted D1‐MSNs modulation. Although both vectors showed robust nigral expression (Figure 5a and Figure S5a, b), their functional impacts on the SNr differed significantly. Clozapine‐N‐oxide (CNO) administration triggered extensive c‐Fos expression in the SNr of hM3Dq‐injected mice, consistent with Gq‐mediated Ca^2+^ signaling. However, rM3Ds‐injected mice showed no increase in nigral c‐Fos+ cells (Figure S5b, c), highlighting the D1‐MSNs‐specific modulation mediated by the rM3Ds effector in our experimental paradigm.

rM3Ds is a Gs‐coupled effector, which activates a cAMP/PKA‐dependent signaling cascade and further stimulates ERK/mTOR pathways [23, 24]. Ribosomal protein S6 (S6), a downstream effector of mTOR, ERK, and PKA signaling, is robustly phosphorylated in activated striatal neurons [25].The phosphorylation level of S6 was significantly increased in the striatum following rM3Ds activation (Figure 5b, c). Correspondingly, slice electrophysiological recordings also revealed increased excitability in D1‐MSNs. Specifically, D1‐MSNs exhibited a higher frequency of induced action potentials and a shorter latency to first AP upon CNO administration, with no significant alterations in resting membrane potential or basal firing rate (Figure 5d–f, and Figure S6a). In accordance with the activation of D1‐MSNs, open‐field testing revealed a significant increase in contralateral rotations following intraperitoneal administration of CNO, compared to saline controls (Figure 5g). However, total travel distance, which indicates locomotor activity, was similar between treatment conditions (Figure 5h). In control mice receiving AAV9R4‐*R9E1‐miniP‐EYFP*, no significant alteration was detected in either S6 phosphorylation or electrophysiological profiles (Figure 5i–m, and Figure S6b). Accordingly, the control mice did not exhibit CNO‐induced rotational behavior (Figure 5n, o). Together, these results demonstrate that the engineered AAV capsid and striatal enhancer enabled targeted chemogenetic activation of the direct pathway, producing robust, pathway‐related motor responses in vivo.

### 2.6 Chemogenetic Activation of the Direct Pathway Alleviates Motor Deficits in a Parkinson's Disease Mouse Model

In PD, loss of SNr dopaminergic neurons leads to reduced activity in the direct pathway of the basal ganglia and overactivation of the indirect pathway, contributing to hallmark motor symptoms including bradykinesia, resting tremor, rigidity, and postural instability [5]. Previous studies have shown that chemogenetic stimulation of D1‐MSNs can alleviate these motor impairments in both rodent and non‐human primate PD models [7, 26]. Here, we sought to test whether our AAV toolkit could generate therapeutic benefits in PD mice by targeting D1‐MSNs.

**FIGURE 6:**
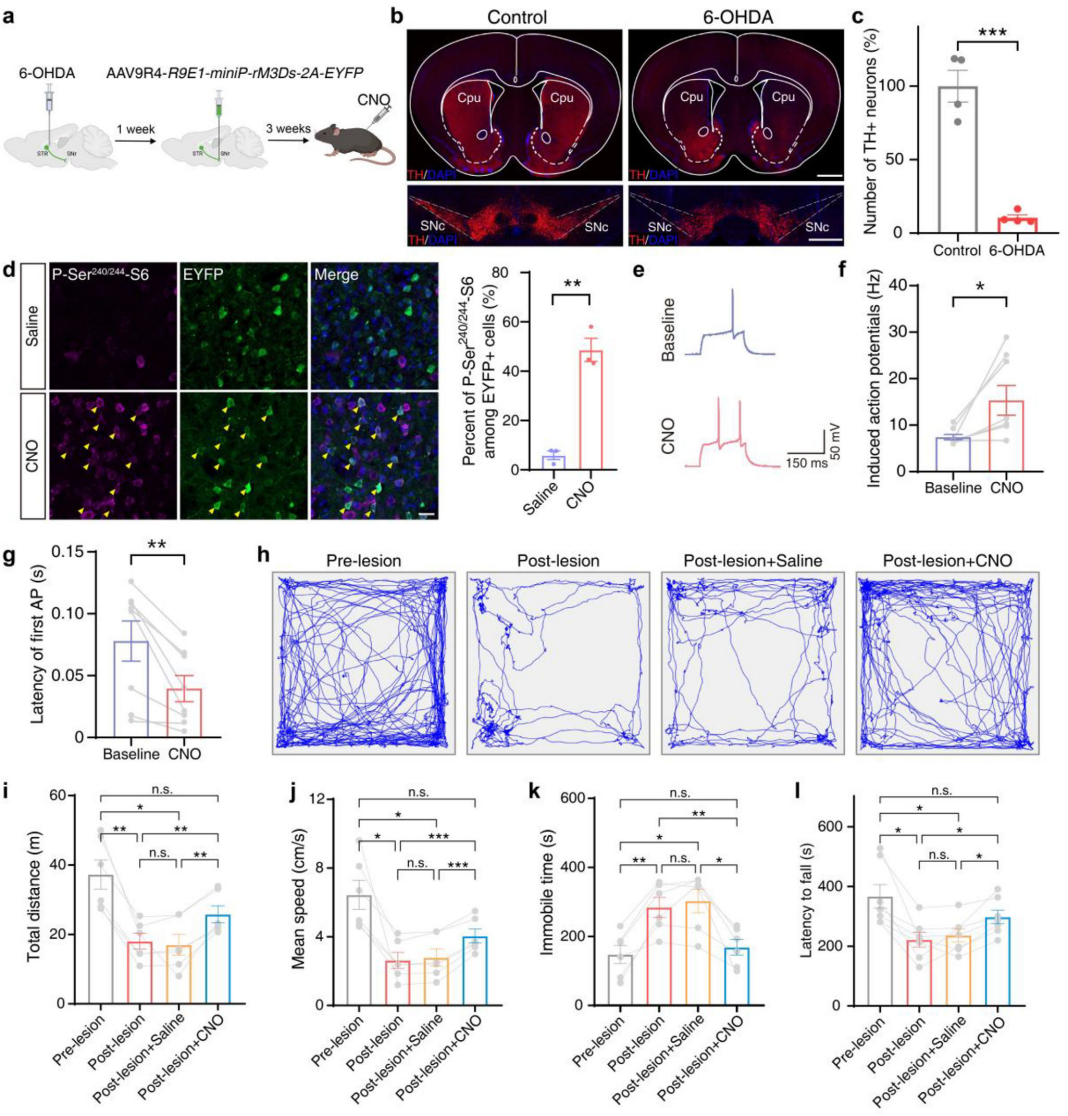
Chemogenetic activation of the direct pathway alleviates behavioral movement deficits in PD mice. (**a)**, Schematic of the generation of parkinsonian mice using 6‐OHDA and selective chemogenetic activation of the direct pathway following nigral AAV9R4‐*R9E1‐miniP‐rM3Ds‐2A‐EYFP* delivery, followed by CNO administration. (**b)**, Representative images showing tyrosine hydroxylase (TH)‐staining of the striatum and the SNc at 1‐week after 6‐OHDA injections. Cpu, caudate‐putamen. SNc, substantia nigra pars compacta. (**c)**, Quantitative analysis of surviving dopaminergic neurons (TH+) in the SNc, numbers were normalized to the control group; n = 4 mice per group, data are represented as mean ± SEM, two‐tailed unpaired *t*‐test, ***p < 0.001. (**d),** Immunofluorescence for P‐Ser^240/244^‐S6 (magenta) of labeled striatal neurons (green) and quantification of P‐Ser^240/244^‐S6+ cells among EYFP+ cells. Arrowheads indicate EYFP cells co‐stained with P‐Ser^240/244^‐S6. Scale bar, 20 µm. n = 3 mice per group, data are represented as mean ± SEM, two‐tailed unpaired *t*‐test, **p < 0.01. (**e–g),** Electrophysiological response to CNO in labeled D1‐MSNs after nigral AAV9R4‐*R9E1‐miniP‐rM3Ds‐2A‐EYFP* injection. Representative traces (**e**), quantification of action potentials (**f**), and latency of first AP (**g**) induced by depolarizing current injection at baseline and after CNO application. n = 8 cells from 4 mice, data are represented as mean ± SEM, two‐tailed paired *t*‐test, *p < 0.05, **p < 0.01. (**h)**, Representative locomotor trajectories during open‐field tests before and after PD modeling, and after saline or CNO treatment in lesioned mice. (**i–l)**, Quantification of total distance traveled (**i**), mean speed (**j**), and time spent immobile (**k**) in an open‐field test and latency to fall off the rotating rod (**l**); n = 6 mice per group, data are represented as mean ± SEM, one‐way ANOVA with Tukey's post‐hoc test, *p < 0.05, **p < 0.01, ***p < 0.001, n.s., not significant.

To establish PD model, we administered 6‐hydroxydopamine (6‐OHDA) bilaterally into the striatum, resulting in near‐complete loss of dopaminergic neurons in the SNc and a marked reduction in dopaminergic innervation of the striatum (Figure 6a–c). One week after the lesions were made, AAV9R4‐*R9E1‐miniP‐rM3Ds‐2A‐EYFP* was bilaterally injected into the SNr to modulate D1‐MSN activity. Three weeks after AAV administration, we observed increased S6 phosphorylation and electrophysiological response following CNO treatment, with no significant alteration in resting membrane potential or basal firing rate (Figure 6d–g, and Figure S6c). Behavioral assessments revealed that, following 6‐OHDA lesions, mice had significantly reduced locomotion and more time spent immobile compared to pre‐lesion baseline measures in the open‐field test, indicating motor impairment in our PD model. These deficits were effectively reversed following CNO administration (Figure 6h–k). In rotarod assays, PD mice showed shortened latency to fall compared to pre‐lesion mice. Following CNO treatment, recovery of motor coordination was observed (Figure 6l).

One of the most common anti‐Parkinsonian medication is levodopa (L‐DOPA) [27], which can also be recapitulated in our models (Figure S7a–e). However, long‐term administration of L‐DOPA can induce dyskinesia. We therefore investigated whether our circuit‐targeted therapy would elicit dyskinesia. We allocated mice lesioned with 6‐OHDA into two groups: 1) L‐DOPA treatment; 2) nigral injection of AAV9R4‐*R9E1‐miniP‐rM3Ds‐2A‐EYFP* followed by CNO treatment. We first evaluated the therapeutic effect of L‐DOPA (Figure S7a–e). We treated mice with L‐DOPA daily for 2 weeks to induce dyskinesia using a standard dose of 3 mg/kg [28]. For the AAV9R4‐*R9E1‐miniP‐rM3Ds‐2A‐EYFP* group, CNO was administered daily for 2 weeks with the dose of 0.3 mg/kg [7]. Dyskinesia was evaluated using abnormal involuntary movement scores (AIMs). We found that L‐DOPA induced robust dyskinetic responses, whereas CNO‐treated animals exhibited minimal dyskinesia (Figure S7f).

The engineered capsid AAV8R12 also efficiently transduced striatal neurons following nigral injection (Figure 2a) [7]. We hypothesized that AAV9R4 could serve as a critical alternative, particularly in scenarios involving pre‐existing immunity against AAV8‐based vectors. To evaluate this potential, we implemented a sequential injection protocol to test cross‐neutralization between these two variants (Figure S8a, b). The results showed that after an initial exposure to AAV8R12, a second dose of the same capsid failed to drive significant EYFP expression in the striatum, likely due to the induction of neutralizing antibodies. In contrast, our engineered AAV9R4 capsid achieved robust D1‐MSN transduction in the same animals (Figure S8c, d). This suggests that AAV9R4 possesses distinct antigenic properties that allow it to escape cross‐neutralization by AAV8‐directed antibodies.

These findings indicate that chemogenetic activation of D1‐MSNs mediated by our AAV toolkit can ameliorate PD‐associated motor deficits, supporting the potential of the toolkit as a gene therapy platform for PD.

## 3 Discussion

There is a critical need in neuroscience to develop precise neuromodulation tools for specific neuronal populations in non‐genetically modified animal models, which would enable both mechanistic studies of neural circuits and therapeutic development for brain disorders [29]. Focusing on D1‐MSNs in the basal ganglia, we have engineered a comprehensive viral toolkit featuring: (1) novel AAV capsids with enhanced retrograde tropism for D1‐MSNs following nigral delivery, and (2) optimized enhancers driving robust transgene expression in striatal neurons. This system achieves efficient and selective D1‐MSN labeling in both rodents and nonhuman primates without requiring genetic modifications. Functionally, we have demonstrated that our toolkit enables precise activation of the direct pathway to induce rotational behaviors in mice. In parkinsonian animals, targeted D1‐MSN stimulation effectively rescued bradykinesia‐like motor deficits, validating both the physiological relevance of this approach and its therapeutic potential for movement disorders. These advanced tools provide researchers with powerful new capabilities for studying basal ganglia function and developing targeted neuromodulation therapies.

In this study, we established an RNA‐based screening strategy for the efficient identification of retrograde AAV capsids. In contrast to conventional DNA‐based approaches [20, 30, 31, 32, 33], RNA‐based methods inherently reduce background noise from non‐functional viral DNA that accumulates in tissues, thereby providing a more accurate reflection of actual transduction events. Additionally, RNA‐based selection may offer greater sensitivity due to the transcriptional enrichment of viral cap gene transcripts. These advantages are exemplified by our successful identification of functional retrograde capsid variants after a single round of selection—an outcome that typically requires two or more rounds in traditional DNA‐based screening protocols. Thus, we envision that this AAV capsid engineering strategy will contribute to future discoveries of additional AAV capsids with distinct tropisms for diverse neural pathways, thereby facilitating retrograde gene therapy across a range of brain disorders with defined circuit dysfunction.

An appealing merit of the AAV9R1 and AAV9R4 variants identified here is their marked retrograde tropism for D1‐MSNs—but not cortical projection neurons—following nigral delivery. In contrast, rAAV2‐retro mainly transduces cortical neurons [20]. The AAV8R12 variant transduces both D1‐MSNs and cortical neurons when transgene expression is driven by the pan‐neuronal *hSyn* promoter [7]. However, despite the improved specificity demonstrated here, the transduction of SNr neurons around injection sites appears to be inevitable. Another layer of control is required to achieve more precise modulation of D1‐MSNs.

Promoters and enhancers are essential for achieving cell‐type‐specific neuronal modulation [34, 35, 36]. While proximal promoters—derived from sequences near transcriptional start sites [37, 38]—have enabled selective gene expression in broad neuronal classes (e.g., neurons vs. glia) or major neuronal types (e.g., excitatory vs. inhibitory neurons), they often lack specificity for finely defined neuronal subtypes [39, 40]. Recent advances in genomic technologies, such as ATAC‐Seq [14] and RNA‐Seq [41], have facilitated the discovery of enhancers with unprecedented subtype specificity [42]. These include enhancer elements targeting PV+ interneurons [43] and distinct subclasses of cortical excitatory and inhibitory neurons [44, 45], as validated in rodent models. In this study, we used a human brain ATAC‐Seq database to identify compact yet potent enhancers capable of driving robust transgene expression in striatal neurons. These *RGS9*‐derived enhancers, when coupled with AAV9R4, achieve highly specific and robust labeling of striatal D1‐MSNs through a synergistic dual‐layer strategy. First, these enhancers are highly active in the striatum. Second, unlike pan‐neuronal promoters (such as *hSyn*), these enhancers leverage the endogenous regulatory logic of the *RGS9* gene, which is naturally enriched in the striatum. This ensures that the transcriptional machinery is primarily activated within striatal tissues, further reducing undesired expression in regions like cortex, SN and STN.

By integrating novel retrograde AAV capsids with MSN‐specific cis‐regulatory elements, our toolkit achieves precise D1‐MSN targeting with minor off‐target transduction of nigral neurons adjacent to the injection site. When combined with rM3Ds, a DREADD that selectively activates the cAMP pathway with negligible effect on nigral neurons, this system further reinforces the precision of D1‐MSN targeting, eliciting a direct pathway‐specific behaviors following systemic CNO administration.

In Parkinson's disease, dopaminergic denervation leads to chronic suppression of D1‐MSN activity and consequent overactivation of basal ganglia inhibitory outputs to thalamocortical circuits, a key neural mechanism underlying PD motor defects [2]. Our retrograde AAV‐based toolkit, combining novel capsids with striatal MSN cis‐regulatory elements, enables targeted D1‐MSN activation that rescues motor deficits in a PD animal model. Importantly, we posit that: (1) the new AAV9‐derived capsid provides a crucial alternative to existing AAV8‐based systems, particularly for patients with AAV8 neutralizing antibodies [46]; (2) sequential administration (AAV8R12 followed by AAV9R1/4) is feasible when therapeutic efficacy wanes and a second dose of AAV8R12 is not advisable due to antibody development; (3) applications of the toolkit extend beyond motor circuits, given the ability to also target D1‐MSNs in the nucleus accumbens due to their molecular similarity to striatal D1‐MSNs [47]. These advances have thus established a versatile platform for both mechanistic studies of basal ganglia function and development of targeted therapies for PD and related disorders.

While our results demonstrate the therapeutic potential of this toolkit in a Parkinsonian model, several hurdles to clinical translation remain. First, unlike conventional gene replacement therapies, chemogenetic interventions require a complex ‘dual‐agent’ regulatory approval process for both the viral vector and the synthetic ligand. Second, the significant increase in brain volume from rodents to humans may affect transduction efficiency and vector distribution, potentially limiting therapeutic scaling. Finally, the immunogenic potential of recombinant AAV capsids and the engineered rM3Ds protein must be considered. The development of humanized DREADD receptors [48] and less immunogenic delivery vehicles will be essential to mitigating these risks in clinical applications.

## 4 Experimental Section

### 4.1 Animals

All animal experiments were performed as described below, and were approved by the Institutional Animal Care and Use Committee (IACUC) at the Shenzhen Institute of Advanced Technology, Chinese Academy of Sciences, following the guidelines stated in the Guide for Care and Use of Laboratory Animals (Eighth Edition, 2011).

Male C57BL/6J mice (8 weeks old) were housed in standard conditions with appropriate humidity and temperature control. Male mice were used for all experiments to eliminate the confounding effects of the female estrous cycle. Mice were randomly assigned to treatment groups.

A cynomolgus monkey (*Macaca fascicularis*) (female, 6 years old, weight 2.5 kg) was housed in an environmentally controlled facility. The animal received a diet consisting of commercial monkey food (Beijing Keao Xieli) supplemented with fresh seasonal fruits, and with free access to water. The monkey was under routine physical examination including careful veterinary monitoring twice daily.

### 4.2 AAV Capsid Library Construction

First, we constructed the AAV library recipient plasmid comprising a CMV promoter and the AAV9 cap gene, flanked by ITRs at both ends. The native splicing donor of Cap9 pre‐mRNA was mutated to its consensus sequence to improve the splicing of Cap9 mRNA. We used the primer GlyNNK‐F (5’‐gacaagtGGCCACAAACCACCAgagcggaNNKNNKNNKNNKNNKNNKNNKgcacaggcgcagaccggctgggtt‐3’; GENEWIZ) which contains 7 NNK repeats flanked by approximately 20 bp, the ends of which overlapped with the targeted plasmid. The anti‐sense sequence of the 3’ end of pGlyNNK‐F, namely pGlyNNK‐R, was used to anneal to pGlyNNK‐F to form dsDNA catalyzed by DNA polymerase. Then, the PCR product (74 bp) was directly assembled with linearized plasmid to generate a DNA library.

The DNA library was then amplified by PCR and co‐delivered to HEK293T cells with helper plasmid and plasmid providing Rep and AAP in trans. Calcium phosphate transfection of HEK293T cells was performed using 24 µg helper plasmid and 12 µg plasmid providing Rep and AAP, and 10 ng library DNA per 15‐cm dish. Production and purification of the AAV virus were conducted using a standard procedure, which was described below.

### 4.3 In Vivo Selection of AAV Capsids

AAVs displaying 7‐mer peptide were delivered to striatum using a stereotaxic apparatus. Tissue samples were prepared 4 weeks after virus injection. To avoid RNA degradation, sample tissues were dissected as soon as possible following extraction of brains. Fresh brain tissue was homogenized using Trizol reagent and total RNA was extracted using a standard protocol and RT‐PCR was then performed as recommended by the manufacturer's instructions. The 7‐mer peptide‐coding sequences were amplified by PCR and then sequenced further using NGS (Illumina NovaSeq system, PE150).

### 4.4 Bioinformatic Analysis of NGS data

Raw image data generated during the sequencing run was processed using CASAVA (v1.8.2) software. This software performs base calling to identify the specific nucleobases and assigns quality scores to each. The resulting sequences were stored in FASTQ (.fq) format, which contains both the nucleotide sequence and its corresponding quality string. During the run, Illumina's internal software applies a “Pass Filter” (PF). A Read was retained only if the signal clusters meet specific quality standards within the first 25 bases; otherwise, the entire Read was discarded.

Raw data often contains adapter sequences or low‐quality bases at the 3’ end, which can introduce bias into downstream biological interpretations. Cutadapt (version 1.9.1) was utilized to trim and filter the raw data, which includes 1) identification and removal of specific primer and adapter sequences; 2) removal of bases from both ends of the sequence if their quality value was lower than 20 (Q20); 3) discarding sequences where the ratio of ambiguous bases (“N”) exceeds 10%. For Paired‐End (PE) sequencing, the DNA fragment was sequenced from both ends, resulting in Read 1 (forward) and Read 2 (reverse) files. Read 1 and Read 2 were then merged into a single continuous sequence based on their overlap region, with Pandaseq (version 2.7). The merged “Clean Reads” were typically converted from FASTQ to FASTA format for subsequent analysis.

Next, the target sequence was precisely extracted by identifying the 10 nucleotides (nt) flanking regions upstream and downstream of the target. After extraction, the workflow performs abundance statistics to count the frequency of each unique sequence within the sample.

Finally, an enrichment score was calculated for each peptide‐encoding sequence—defined as the ratio of its frequency in the striatal sample to that in the initial AAV pool. The sequences were then ranked by enrichment score. The enriched peptides with a cutoff ≥40 were clustered by similarity (≥70%). The consensus motif of each cluster was generated.

### 4.5 Structure Prediction by AlphaFold3

Sequences of VR‐VIII from AAV9 and AAV9R1 ∼ R5 were inputted into AlphaFold3 (Google DeepMind) to predict their structures, with default parameters set. Then, the structures were visualized and annotated using PyMOL (Schrödinger).

### 4.6 Screening of Putative Enhancers

First, we screened striatum marker genes in the Allen Brain Atlas (http://mouse.brain‐map.org/), and identified *RGS9*, which was highly expressed in the striatum but not in other brain regions. Next, we analyzed Roussos lab Brain Open Chromatin Atlas (BOCA35) and discovered four short open chromatin regions (OCRs) within the *RGS9* gene locus that were specific to the striatum and nucleus accumbens [14, 15]. Finally, the sequences of candidate OCRs were PCR‐amplified from human genomic DNA, cloned into rAAV vectors, and their activity tested following nigral injections in adult mice.

### 4.7 Plasmid Construction

Enhancers upstream of the miniCMV promoter (*miniP*) were cloned into the pAAV‐*hSyn‐EYFP* vector derived from pAAV‐*hSyn‐EGFP* (Addgene, 50465) to replace the *hSyn* promoter, with appropriate restriction enzyme combinations. To generate pAAV‐*R9E1‐miniP‐rM3Ds‐2A‐EYFP*, *R9E1‐miniP* was cloned into pAAV‐*G88P7‐rM3Ds‐2A‐EYFP* (Addgene, 213970) to replace the *G88P7* promoter. Constructed plasmids were confirmed following Sanger sequencing. To generate pAAV‐*R9E1‐miniP*‐DIO‐tdTomato, a DIO‐tdTomato cassette was subcloned into pAAV‐*R9E1‐miniP*‐EYFP to replace EYFP by restriction enzyme digestion. To generate pAAV‐*R9E1‐miniP*‐hM3Dq‐2A‐EYFP, a hM3Dq fragment was subcloned into pAAV‐*R9E1‐miniP‐rM3Ds‐2A‐EYFP* to replace *rM3Ds* by restriction enzyme digestion.

### 4.8 AAV Production

For individual recombinant AAVs packaging, the AAV Rep‐Cap plasmid (rAAV2‐retro (Addgene, 81070), AAV8R12 (Addgene, 213968), AAV9 (Addgene, 112865), PHP.eB (Addgene, 103005), AAV9R1∼R5, the AAV vector plasmids and the pAdDeltaF6 helper plasmid (Addgene, 112867) were co‐transfected into HEK293T cells with calcium phosphate.

Cells were collected with resuspension buffer (150 mM NaCl, 100 mM Tris‐HCl, pH 8.0) 72 h after transfection. The virus particles were released through repeated freeze/thaw cycling, followed by purification and concentration using 100 kDa ultrafiltration tubes (100 KD, Merck Millipore). Viral titer quantification was performed by extracting viral DNA and conducting qPCR with primers targeting the ITR regions. Finally, the purified viral particles were treated with Proteinase K and stored at −80°C until further use.

### 4.9 AAV Injection

#### 4.9.1 Mice

Animals were anesthetized via intraperitoneal injection of pentobarbital sodium (Sigma, P3761) and secured in a stereotactic frame (RWD Instruments) with ear bars and a nose clamp to immobilize the head. After exposing the skull via a midline scalp incision, bregma and lambda were aligned to ensure a flat skull position. A small craniotomy was made above the SNr (from bregma: AP −3.4 mm, ML 1.3 mm, DV −4.8 mm) and 200 nL of viral vectors (1×10^13^ VG/mL) were delivered via a 10‐µL 33‐Gauge Hamilton syringe at a rate of 20 nL/min. For AAV delivery in striatum (AP +0.5 mm, ML 1.7 mm, DV −3.5 mm), 500 nL of viral vectors (3×10^12^ VG/mL) were delivered at a rate of 50 nL/min. For retrograde tracing of AAV9R4 in other brain regions, AAV9R4‐hSyn‐mCherry was injected to the M1 (AP: +2.1 mm, ML: −2.0 mm, DV: −1.7 mm), the medial prefrontal cortex (mPFC; AP: +1.54 mm, ML: −0.3 mm, DV: −2.5 mm), the dorsolateral striatum (DLS; AP: +0.5 mm, ML: −2.25 mm, DV: −3.3 mm), the dorsal lateral geniculate nucleus (dLGN; AP: −2.06 mm, ML: −2 mm, DV: −2.8 mm), and the superior colliculus (SC; AP: −3.4 mm, ML: −0.6 mm, DV: −1.8 mm). Injection volumes were 200 nL for the M1, the mPFC, the dLGN and the SC, and 300 nL for the striatum. The virus titers were 1×10^13^ VG/mL. The needle was left in place for 10 min post‐injection to prevent backflow. For intravenous injection, 100 nL of viral vector (1×10^11^ VG per animal) was delivered via the retro‐orbital venous sinus using an insulin syringe. Brain tissue sampling or behavioral experiments were performed at least 3 weeks after virus injection.

#### 4.9.2 Monkey

The injection into the monkey was performed as previously described [7]. All surgical instruments were sterilized or disinfected before use. The animal was fasted for 12 h prior to surgery. The animal was given atropine (0.05 mg/kg, intramuscular) before ketamine administration (15 mg/kg, intramuscular) to induce sedation just before surgery. Endotracheal intubation was then performed and general anesthesia with 1%–3% isoflurane in 100% O_2_ was maintained throughout. Vital signs were monitored throughout the procedure. The head was fixed in a primate stereotaxic frame (Kopf Instruments) and an MRI‐compatible guiding grid with regularly arranged holes was fixed in the skull. This grid was filled with the contrast agent Vitamin E to allow the holes to be imaged under T1‐weighted MRI scans and served as marker points to locate the coordinates for the SN. A total volume of 27 µL of virus was unilaterally injected in nine sites across the right SNr (3 µL/sites) at a speed of 300 nL/min using 10‐µL 33‐Gauge Hamilton syringe. The needle was held in place for 10 min post‐injection to minimize reflux. The scalp was sutured in layers, and a topical antibiotic ointment was applied. The animal recovered in a temperature‐controlled environment with continuous monitoring until fully awake. Postoperative analgesia and antibiotics were administered for 48–72 h. Brain tissue sampling was performed 6 weeks after virus injection.

### 4.10 6‐hydroxydopamine (6‐OHDA) Injection

A PD mouse model was generated using 6‐OHDA. Thirty minutes prior to 6‐OHDA administration, mice received an intraperitoneal injection of desipramine (25 mg/kg, Sigma). The stereotactic injection procedure was similar to the viral vector injection as described above. Briefly, bilateral striatal injections were performed using the following coordinates relative to bregma: AP 0.5 mm, ML ±1.5 mm, DV −3.2 mm. A volume of 800 nL 6‐OHDA (5 mg/ml in 0.02% ascorbic acid‐saline) was injected into each side of striatum at a rate of 100 nL/min. One week after 6‐OHDA lesions were generated, animals received AAV injections, and brain tissue sampling or behavioral tests were conducted 3 weeks after AAV injections.

### 4.11 Brain Slice Electrophysiological Recordings

Three weeks after‐AAV injection, mice from different groups were anesthetized, followed by cardiac perfusion with pre‐oxygenated (95% O_2_ + 5% CO_2_) and ice‐cold (0–4°C) NMDG‐based cutting solution (in mM): 92 NMDG, 2.5 KCl, 25 NaHCO_3_, 1.25 NaH_2_PO_4_, 4.5 D‐Glucose, 20 HEPES, 5 L‐ascorbic acid, 3 Na‐pyruvate, 2 Thiourea, 10 MgSO_4_, 0.5 CaCl_2_ (pH 7.25 ± 0.05, osmolarity 305 ± 5 mOsm/kg). Following sufficient perfusion, coronal brain slices (300 µm thickness) were prepared using a vibratome (Leica VT1200S). After sectioning, the slices were transferred to oxygenated artificial cerebrospinal fluid (aCSF) at 34°C for 30 min, and then placed at room temperature for at least 30 min. The aCSF contains (in mM): 125 NaCl, 1.25 KCl, 25 NaHCO_3_, 1.25 KH_2_PO_4_, 25 D‐Glucose, 2 CaCl_2_ and 1 MgCl_2_, 2 Na‐pyruvate, 3 Myo‐inositol and 0.4 mM L‐ascorbic acid (pH 7.35 ± 0.05, osmolarity 305 ± 5 mOsm/kg).

The brain slices were moved into the recording chamber and continuously perfused with oxygenated aCSF at a rate of 3−4 mL/min. Glass microelectrodes (OD = 1.5 mm, VitalSense B15014F) were pulled into recording glass microelectrodes using an horizontal puller (RWD life science, HF‐3030B) to a final tip resistance of approximately 6 MΩ. The recording electrodes were filled with K^+^‐based internal solution containing (in mM): 135 KMeSO_3_, 10 KCl, 10 HEPES, 5 MgATP, 0.5 NaGTP, 1 EGTA, pH was adjusted to 7.2 ± 0.1 using KOH and the osmolarity was adjusted to 300 ± 5 mOsmol/L using KMeSO_3_.

The action potential rheobase was determined by injecting a series of 150‐ms depolarizing current steps ranging from 0 to +400 pA (with a step of +10 pA, an interval of 10,000 ms). The rheobase was defined as the minimal depolarizing current injection required to elicit a single action potential (AP). Baseline induced action potentials was recorded by applying the rheobase stimulus (150‐ms duration, 10,000 ms inter‐stimulus interval) no fewer than 20 times. Following baseline recording, the perfusion solution was switched to aCSF containing 10 µM CNO. After a 3‐min stabilization period in CNO‐containing aCSF, the identical 150‐ms rheobase stimulus was reapplied. The mean action potential firing frequency was calculated separately for the baseline period and following CNO perfusion. Data analysis was performed using Clampfit 10.5 software (Molecular Devices).

### 4.12 Behavioral Measurements

#### 4.12.1 Open‐Field Test

Mice were acclimated to the testing room and experimenter for 3 days and underwent 1 day of habituation to the test arena, which had a length, width and height of 50 × 50 × 50 cm. On the test day, 30 min after injection of either saline, CNO (Hello Bio, 0.3 mg/kg) or L‐DOPA (Aladdin, 3 mg/kg) plus benserazide (MCE, 12 mg/kg), the animals were placed into the test box. Video was recorded for 10 min. Between trials, the arena was cleaned with 70% ethanol to eliminate odor cues. Each mouse was tested only once to avoid habituation effects. The total distance traveled, the number of rotations and the time spent immobile were then analyzed using Anymaze software.

#### 4.12.2 Rotarod Test

The rotarod test was performed using an accelerating rotating rod apparatus (Xinruan). After 3 days of habituation to the environment and operator, the mice were placed on a suspended rod apparatus and trained at speeds of 10, 20, 30 and 40 rpm/min for 2 consecutive days. Each training session used one speed and lasted 10 min. Between consecutive speed sessions, animals were allowed a 15‐minute recovery period. Each mouse was trained on this sequence of speeds each day. On the test day, each mouse performed 3 test trials (10, 20, 30 and 40 rpm/min, test duration: 10 min) with a 15‐minute inter‐trial rest interval. The mean latency to fall was recorded and used for further calculations.

### 4.13 Abnormal Involuntary Movements (AIMs)

In the L‐DOPA treatment group, L‐DOPA was administered intraperitoneally daily at doses of 3 mg/kg, in combination with benserazide at 12 mg/kg for 2 weeks [49]. In the AAV9R4‐*R9E1‐miniP‐rM3Ds‐2A‐EYFP* combined with CNO treatment group, CNO (0.3 mg/kg) was administered intraperitoneally daily for 2 weeks. On the final day of treatment, mice were habituated to the testing environment and handling procedures, then placed individually in a transparent beaker and allowed to move freely. Video recording began 20 min after L‐DOPA or CNO treatment and lasted for 140 min. AIMs were scored every 20th minute using a previously established scale [28, 50] as follows: 0 = no dyskinesia; 1 = occasional signs of dyskinesia; 2 = frequent dyskinetic movements, present for >50% of the observation period; 3 = continuous dyskinetic movements, but interruptible by mild external stimuli; 4 = continuous dyskinesia, uninterrupted by mild external stimuli.

### 4.14 Immunofluorescence Staining

Animals were deeply anesthetized and transcardially perfused with PBS (50 mL for mice; 1 L for monkey) and 4% paraformaldehyde (50 mL for mice; 1 L for monkey). The brains were extracted, post‐fixed overnight, and then dehydrated with 30% sucrose solution at 4°C. After being embedded in OCT (Sakura), brain sections (40‐µm thickness for mice; 50‐µm thickness for the monkey) were obtained using a cryostat (Leica, CM1950). For c‐Fos or S6 phosphorylation detection, CNO or saline were administrated 30 min before the animals were perfused.

Brain slices were permeabilized and blocked with PBS containing 5% bovine serum albumin (BSA) and 0.3% Triton X‐100 for 1 h at room temperature. The slices were then incubated with anti‐RFP (Abcam), anti‐GFP (Abcam) or anti‐TH (Abcam), anti‐NeuN (Abcam), anti‐PV (Swant), anti‐SST (Abcam), anti‐ChAT (Sigma Aldrich), anti‐c‐Fos (CST), anti‐p‐Ser^240/244^‐S6 (CST) diluted to 1:500 in PBS containing 5% BSA at 4°C overnight. After thorough washing, the sections were then incubated with diluted Alexa Fluor 488 goat anti‐chicken IgG antibody, Alexa Fluor 594 donkey anti‐goat IgG, Alexa Fluor 594 donkey anti‐rabbit IgG, or Alexa Fluor 594 donkey anti‐mouse IgG antibody (Thermo Fisher) accordingly at room temperature for 1 h. Cell nuclei were counterstained with DAPI (Sigma). For S6 phosphorylation staining, NaF (0.1 mM) was included in all buffers and incubation solutions. Images were visualized using a slide scanner (Olympus, BX61VS) and a confocal microscope (Zeiss, LSM880). When counting labeled cells throughout the entire brain, every eighth section was analyzed. Therefore, the number of labeled cells counted in each section was multiplied by eight to estimate the total number of labeled cells per animal.

### 4.15 In Situ Hybridization

Digoxigenin (DIG)‐labeled cRNA probes (riboprobes) for mice Drd1/Drd2 or macaques DRD1/DRD2 were prepared as previously described [7]. Fresh‐frozen brain tissues were fresh‐frozen in OCT (Sakura) and then sectioned at 40–50 µm thickness using a cryostat. Sections were incubated with proteinase K and then acetylated with 0.25% acetic anhydride in 0.1 M triethanolamine for 10 min at room temperature. Sections were then hybridized with DIG‐labeled cRNA probes at 56°C for 15–18 h. Following hybridization, sections were washed twice in 0.2X SSC at 65°C for 40 min to remove unbound probes. Sections were then incubated with peroxidase (POD)‐conjugated anti‐DIG antibodies (Roche) at 37°C for 1 h, and then treated with the TSA‐plus kit (Perkin Elmer). Sections were then incubated with anti‐GFP antibody (Abcam) at 4°C overnight and finally with Alexa Fluor 488 goat anti‐chicken IgG antibody (Thermo Fisher) at room temperature for 2 h. DAPI (Sigma) was also applied to counterstain the nuclei.

### 4.16 Statistical Analysis

The data were expressed as the mean ± standard error and analyzed using GraphPad Prism (version 9.0). Multiple group comparisons were conducted using the paired t‐test, unpaired t‐test, one‐way ANOVA, Tukey's test, and Dunnett's test, as appropriate. All *t*‐tests were performed as two‐tailed. Statistical significance was set at a *p*‐value <0.05. All statistical tests used were indicated in the figure legends.

## Author Contributions

T.L., Y.J., Yuantao Li, Y.C., and Z. Lu. designed the project. T.L., Z.H., and Y.Z. constructed the AAV capsid library and performed the screening in vivo. J.Z., H.L., Q.L., L.Y, L.L., Z.Liu., and Z.Y. performed computational analysis with input from T.L., Z.H., Y.Z., and J.Z. Z.H. and Yanglei Li performed the animal behavioral experiments. T.L., Z.Lu, Yuantao Li., Y.J., and Y.C. wrote the manuscript with input from all authors.

## Conflicts of Interest

Z.H., and T.L. are co‐inventors on a provisional patent that is being filed for this technology. The application number is PCT/CN2025/134593.

## Supporting information




**Supporting File**: advs75954‐sup‐0001‐suppMat.doc.

## Data Availability

The data that support the findings of this study are available in the supplementary material of this article.
