# Supplementary material for: A Toolkit for Targeted Neuromodulation of Striatal Direct Pathway Neurons Rescues Parkinsonian Motor Deficits in Mice
Source: Adv Sci (Weinh). 2026 Jun 22:e19665. Online ahead of print. doi: 10.1002/advs.202519665 (PMC13337097; doi:10.1002/advs.202519665)
Supplement: Supplementary file 1 — Supporting File: advs75954‐sup‐0001‐suppMat.doc. [file ADVS-9999-e19665-s001.doc]

A Toolkit for Targeted Neuromodulation of Striatal Direct Pathway Neurons Rescues Parkinsonian Motor Deficits in Mice

Zexuan Hong, Yujing Zhang, Junjiao Zhang, Hanhe Liu, Qiwei Liu, Yanglei Li, Lixin Yang, Lixia Li, Zhongjie Liu, Zhen Yuan, Zhonghua Lu, Yefei Chen*, Yuantao Li*, Yuwu Jiang*, and Taian Liu*

Z. Hong, Yuantao Li

Department of Anesthesiology, Shenzhen Maternity and Child Healthcare Hospital, Women and Children's Medical Center, Southern Medical University, Shenzhen 518027, China
E-mail: szmchlyt@smu.edu.cn

Z. Hong, Y. Zhang, H. Liu, Q. Liu, Yanglei Li, L. Yang, Z. Lu, T. Liu
Research Center for Primate Neuromodulation and Neuroimaging, Institute of Biomedical and Health Engineering, Shenzhen Institutes of Advanced Technology, Chinese Academy of Sciences, Shenzhen 518055, China

Shenzhen Key Laboratory for Molecular Biology of Neural Development, Shenzhen Technological Research Center for Primate Translational Medicine, Shenzhen-Hong Kong Institute of Brain Science, Shenzhen Institutes of Advanced Technology, Chinese Academy of Sciences, Shenzhen 518055, China

E-mail: [ta.liu@siat.ac.cn](mailto:ta.liu@siat.ac.cn)

J. Zhang, Y. Jiang

Children’s Medical Center, Peking University First Hospital, Beijing 100034, China

E-mail: jiangyuwu@bjmu.edu.cn

L. Li, Z. Liu

Department of Anesthesia, Affiliated Shenzhen Children's Hospital of Shantou University Medical College, Shenzhen, Guangdong, China

Y. Zhang, Z. Yuan

Faculty of Health Sciences, University of Macau, Macau SAR 999078, China

Z. Lu, Y. Chen, T. Liu

Department of Neurology, The Second Affiliated Hospital and School of Brain Science and Brain Medicine, Zhejiang University School of Medicine, Hangzhou 310058, China

Dreambrook Research Institute of Brain Disorders, Hangzhou 311121, China

E-mail: [yf.chen@siat.ac.cn](mailto:yf.chen@siat.ac.cn)

Yuantao Li

Biomedical Research Institute, Hubei University of Medicine, Shiyan 442000, China


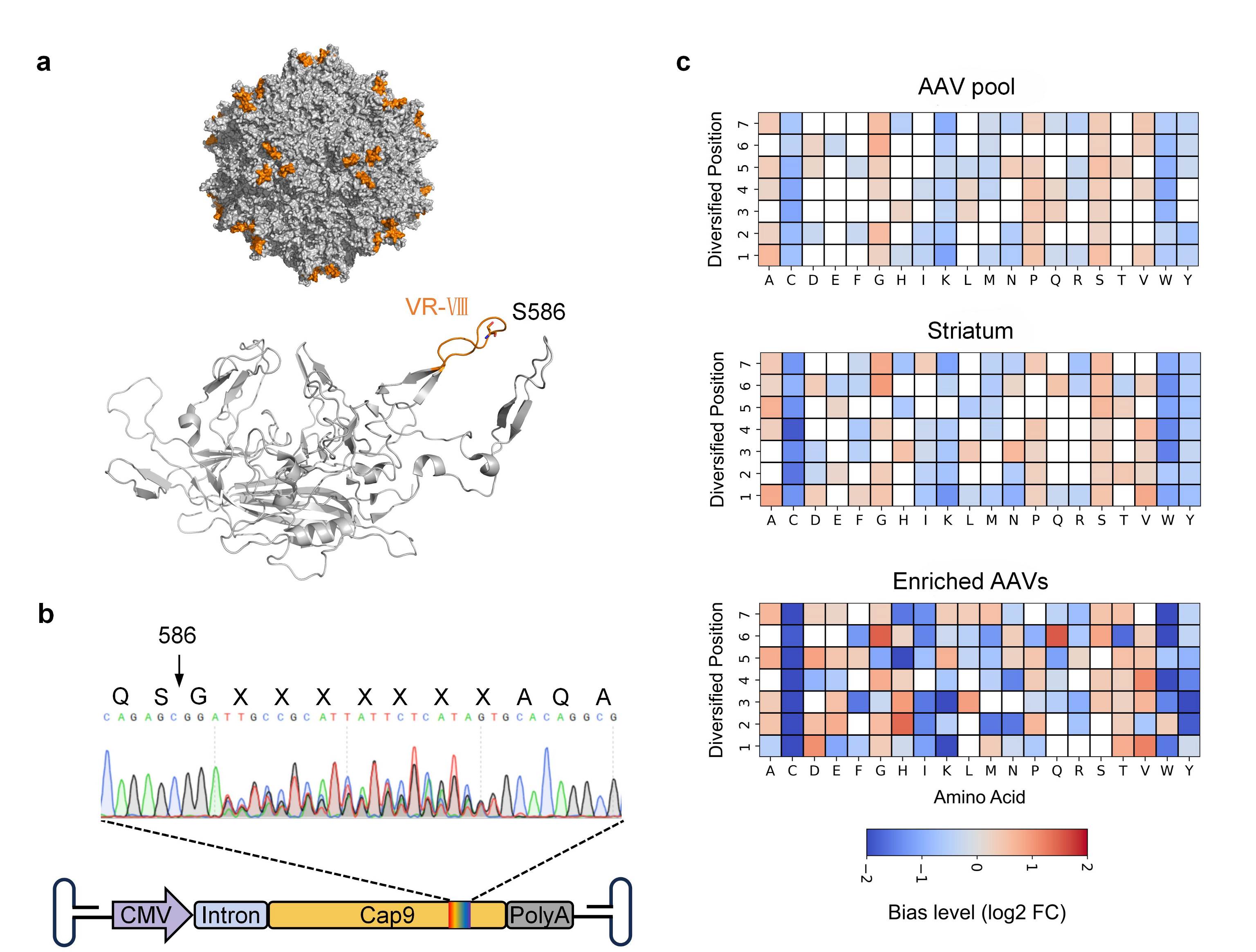


**Supplementary Figure 1.** AAV9 library construction and evolution in mouse brain.

**a**, Structural reconstitution of AAV9 viral particle (top) and VP3 cap protein (bottom). The VR-Ⅷ region amenable to insertion is highlighted in orange.

**b**, Sanger sequencing of the AAV9 capsid DNA library with a random 7-mer peptide insertion at S586.

**c**, Heat maps showing the frequency change of a given amino acid in each position of 7-mer peptides, relative to theoretical probability of NNK codons. These peptides are recovered from original AAV library, striatum, and enriched AAVs, respectively.


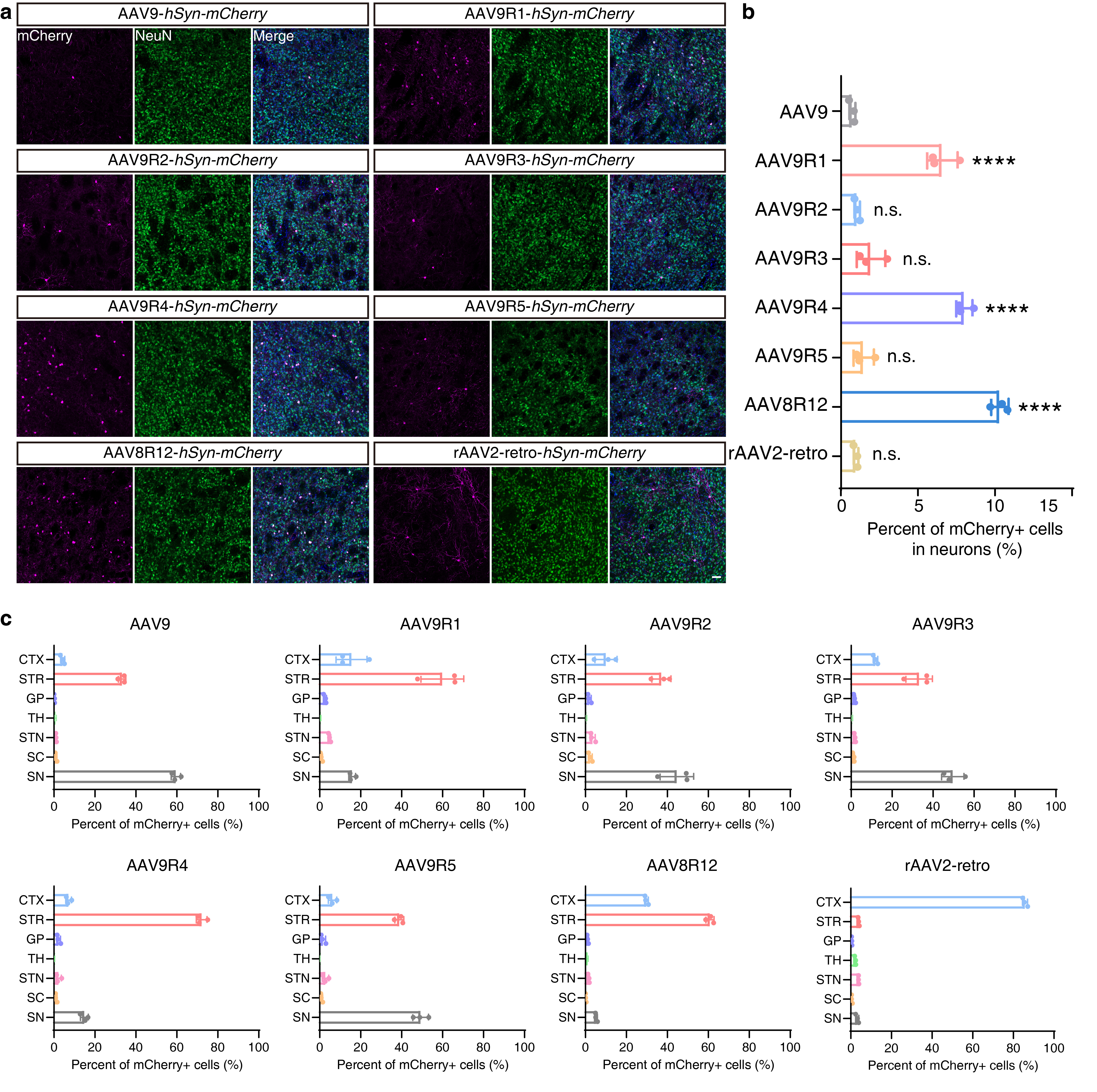


**Supplementary Figure 2.** Transduction patterns of selected AAV9 capsid variants following nigral injections.

**a,b,** Representative images (**a**) of retrogradely labeled striatal neurons (magenta) with NeuN staining (green) and quantitation of mCherry+ cells amongst NeuN+ cells (**b**). Scale bar, 50 μm. n=3 mice per group, data are represented as mean ± SEM, one-way ANOVA with post-hoc Tukey’s test. Statistical comparisons are with the AAV9 group, *****p* < 0.0001, n.s., not significant.

**c**, Percentages of labeled neurons by nigral AAV injection in different brain regions across the brain. CTX: cortex, STR: striatum, GP: globus pallidus, TH: thalamus, STN: subthalamic nucleus, SC: superior colliculus, SN: substantia nigra. n=3 mice per group, data are represented as mean ± SEM.


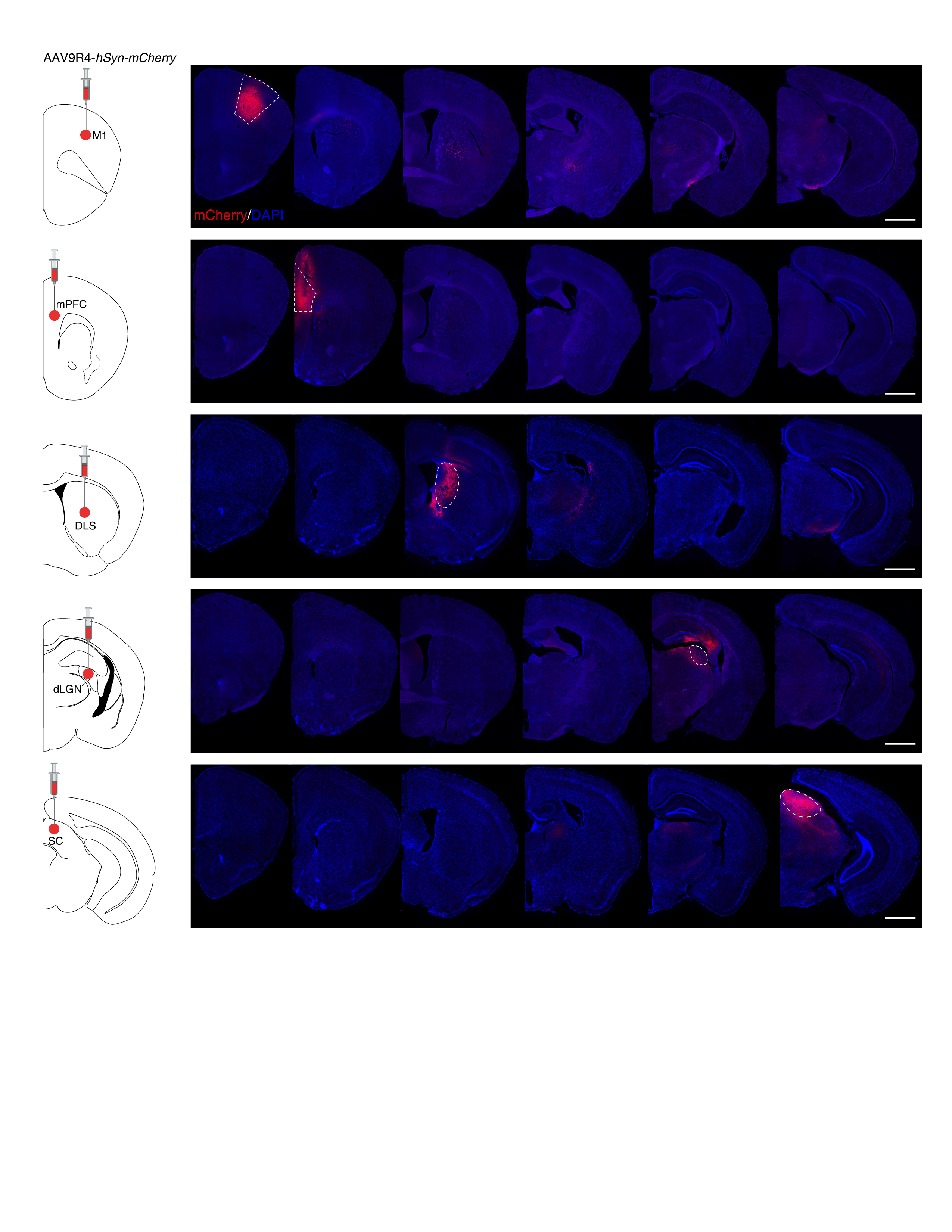


**Supplementary Figure 3.** Scarce retrograde labeling by AAV9R4 after injections in non-SNr brain regions.

Representative images showing expression pattern after injection of AAV9R4-*hSyn-mCherry* to brain regions including primary motor cortex (M1), medial prefrontal cortex (mPFC), dorsolateral striatum (DLS), dorsal lateral geniculate nucleus (dLGN), and superior colliculus (SC). Scale bar, 1 mm. Dashed lines mark the injection sites.


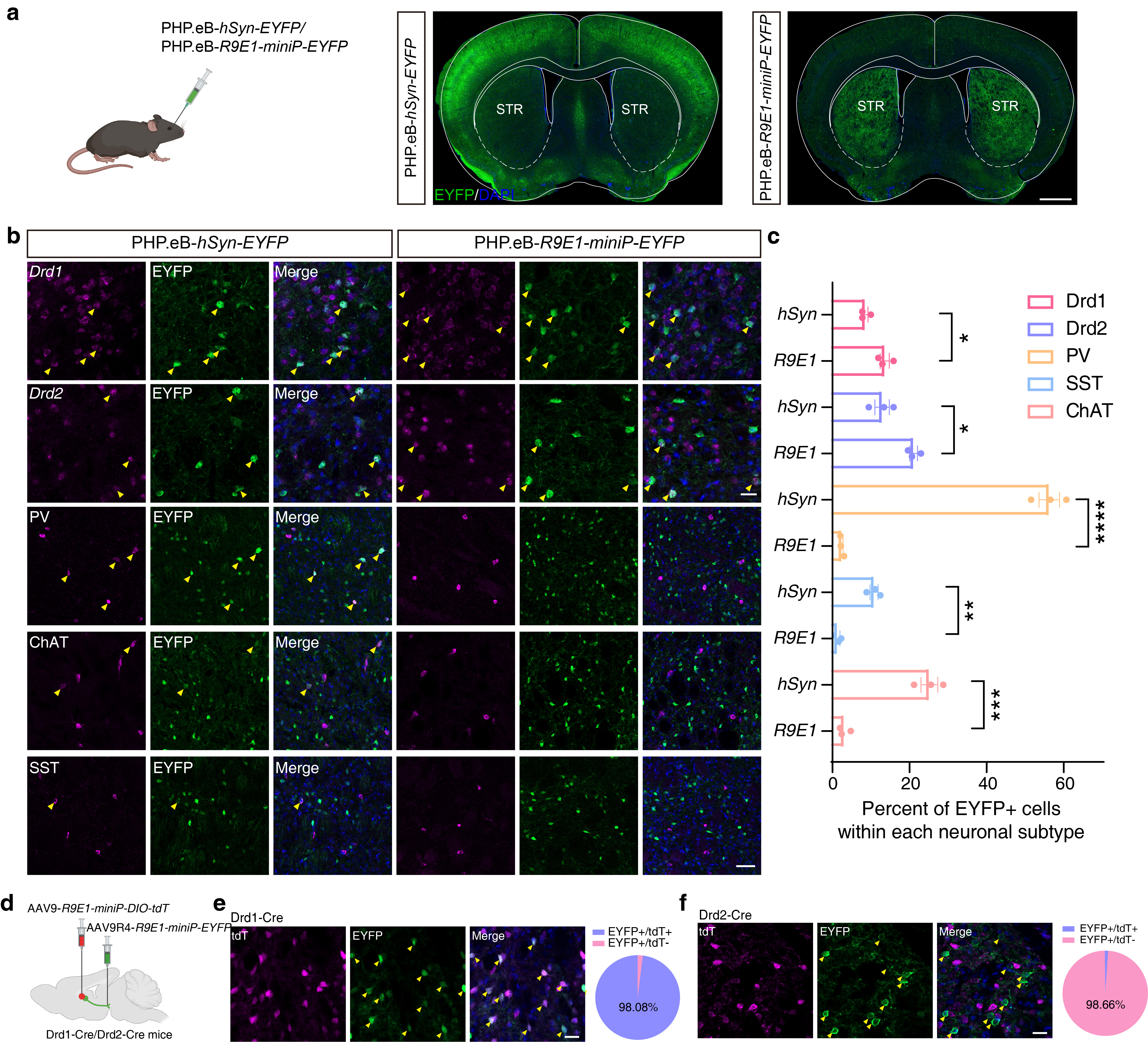


**Supplementary Figure 4.** Characterization of labeling specificity of PHP.eB-*R9E1-miniP-EYFP* after intravenous delivery and AAV9R4-*R9E1-miniP-EYFP* after nigral injection.

**a,** Representative images showing expression pattern after intravenous delivery of PHP.eB-*R9E1-miniP-EYFP* or PHP.eB-*hSyn*-*EYFP.* Scale bar, 1 mm.

**b,** Co-staining of transduced neurons (EYFP, green) with *Drd1*, *Drd2*, parvalbumin (PV), somatostatin (SST), or ChAT (magenta). Arrowheads indicate double+ cells. Scale bars, 20 μm (*Drd1* and *Drd2*), 50 μm (PV, SST, and ChAT).

**c,** Quantitation of *Drd1+*, *Drd2+*, PV+, SST+, and ChAT+ cells amongst EYFP+ cells in the striatum. n=3 mice per group, data are represented as mean ± SEM, two-tailed unpaired *t*-test, **p* < 0.05, ***p* < 0.01, ****p* < 0.001, *****p* < 0.0001.

**d,** Retrograde labeling by stereotaxic injections of AAV9R4-*R9E1-miniP-EYFP* into the SNr and AAV9-*R9E1-miniP-DIO*-*tdTomato* into the striatum in Drd1-Cre or Drd2-Cre mice.

**e,f,** Left, Retrograde labeling of striatal neurons (EYFP, green, arrowheads) and Cre-driven tdTomato expression (tdT, magenta) in Drd1-Cre (**e**) or Drd2-Cre (**f**) mice. Right, quantitation of tdT+ and tdT- cells amongst EYFP+ cells in the striatum. Scale bars, 20 μm. n=3 mice per group.


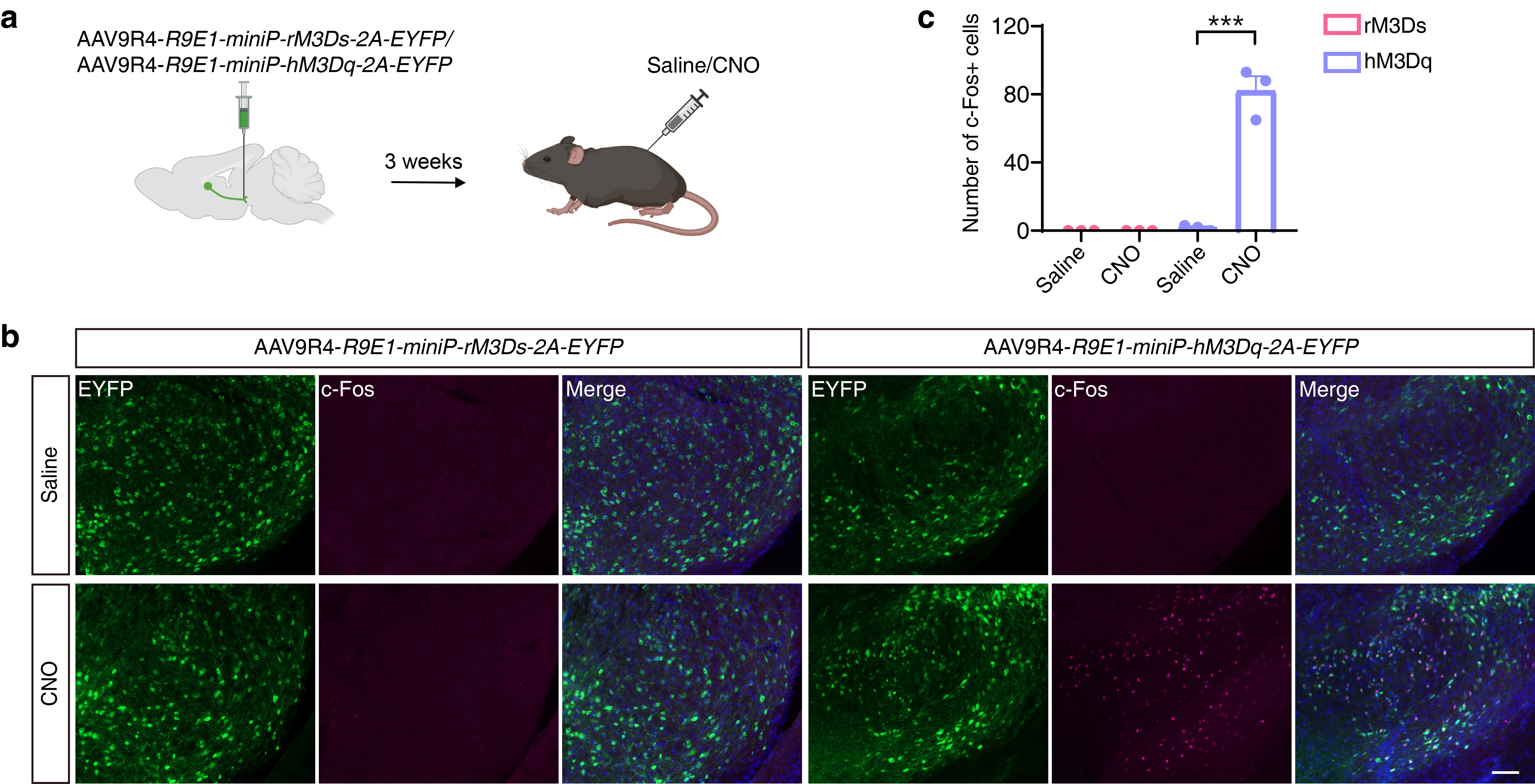


**Supplementary Figure 5.** Nigral injection of AAV9R4-*R9E1-miniP-rM3Ds-2A-EYFP* in mice did not activate nigral neurons.

**a,** Schematic showing SNr injection of AAV9R4-*R9E1-miniP-rM3Ds-2A-EYFP* or AAV9R4-*R9E1-miniP-hM3Dq-2A-EYFP* (control).

**b,** Representative images showing AAV transduced cells (EYFP, green) and c-Fos (magenta) staining in the SNr after CNO delivery. Scale bar, 100 μm.

**c,** Quantitation of cFos+ cells in SNr. n=3 mice per group, data are represented as mean ± SEM, two-tailed unpaired *t*-test, ****p* < 0.001.


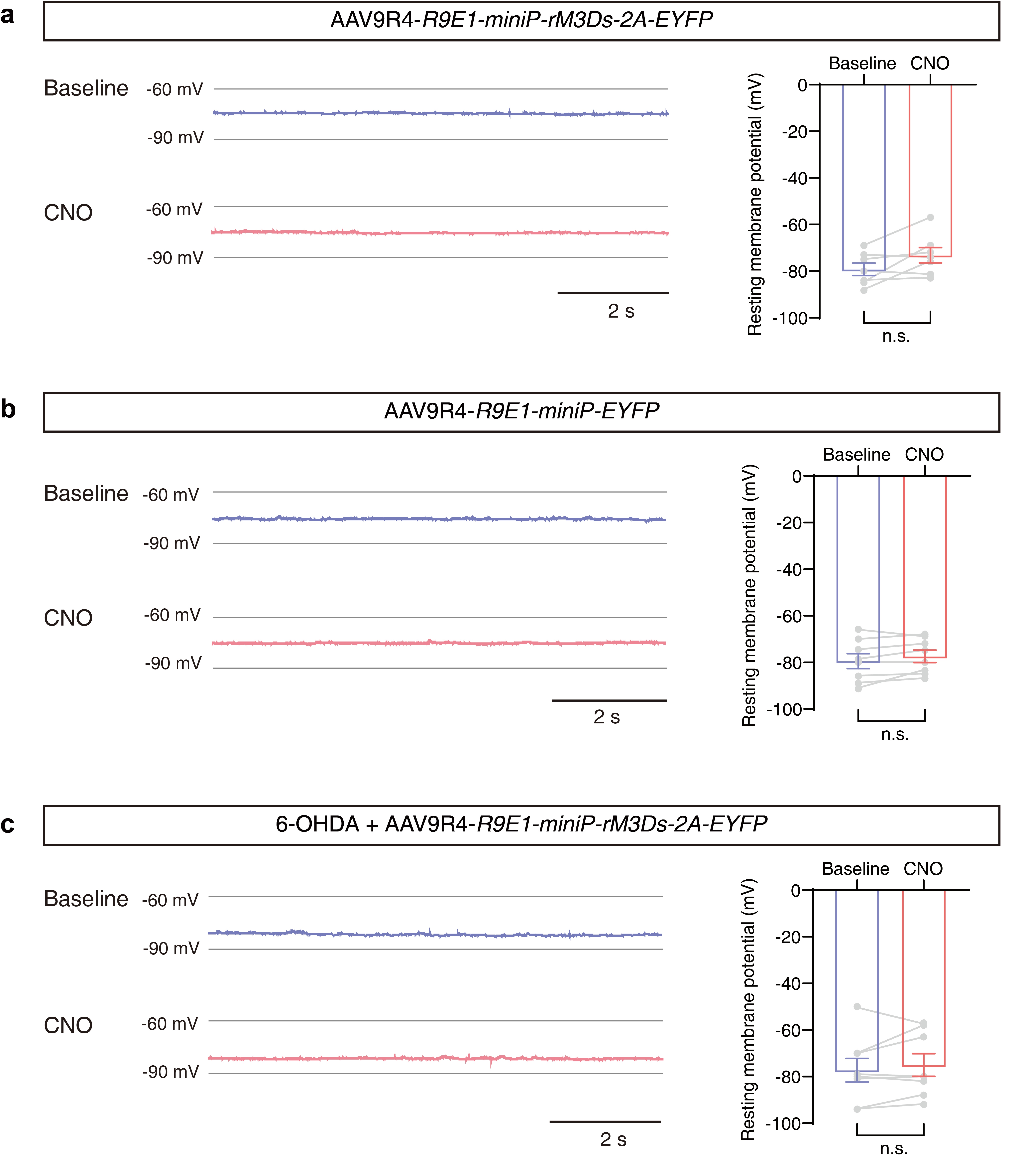


**Supplementary Figure 6.** Chemogenetic activation did not affect basal activity in striatal MSNs.

**a,** Basal activity without current injection and resting membrane potential were recorded in mice received nigral AAV9R4-*R9E1-miniP-rM3Ds-2A-EYFP* injections before and after CNO incubation. n = 7 cells from 6 mice, data are represented as mean ± SEM, two-tailed paired *t*-test, n.s., not significant.

**b,** Basal activity without current injection and resting membrane potential were recorded in mice received nigral AAV9R4-*R9E1-miniP-EYFP* injections before and after CNO incubation. n = 8 cells from 6 mice, data are represented as mean ± SEM, two-tailed paired *t*-test, n.s., not significant.

**c,** Basal activity without current injection and resting membrane potential were recorded in 6-OHDA lesioned mice received nigral AAV9R4-*R9E1-miniP-rM3Ds-2A-EYFP* injections before and after CNO incubation. n = 8 cells from 4 mice, data are represented as mean ± SEM, two-tailed paired *t*-test, n.s., not significant.


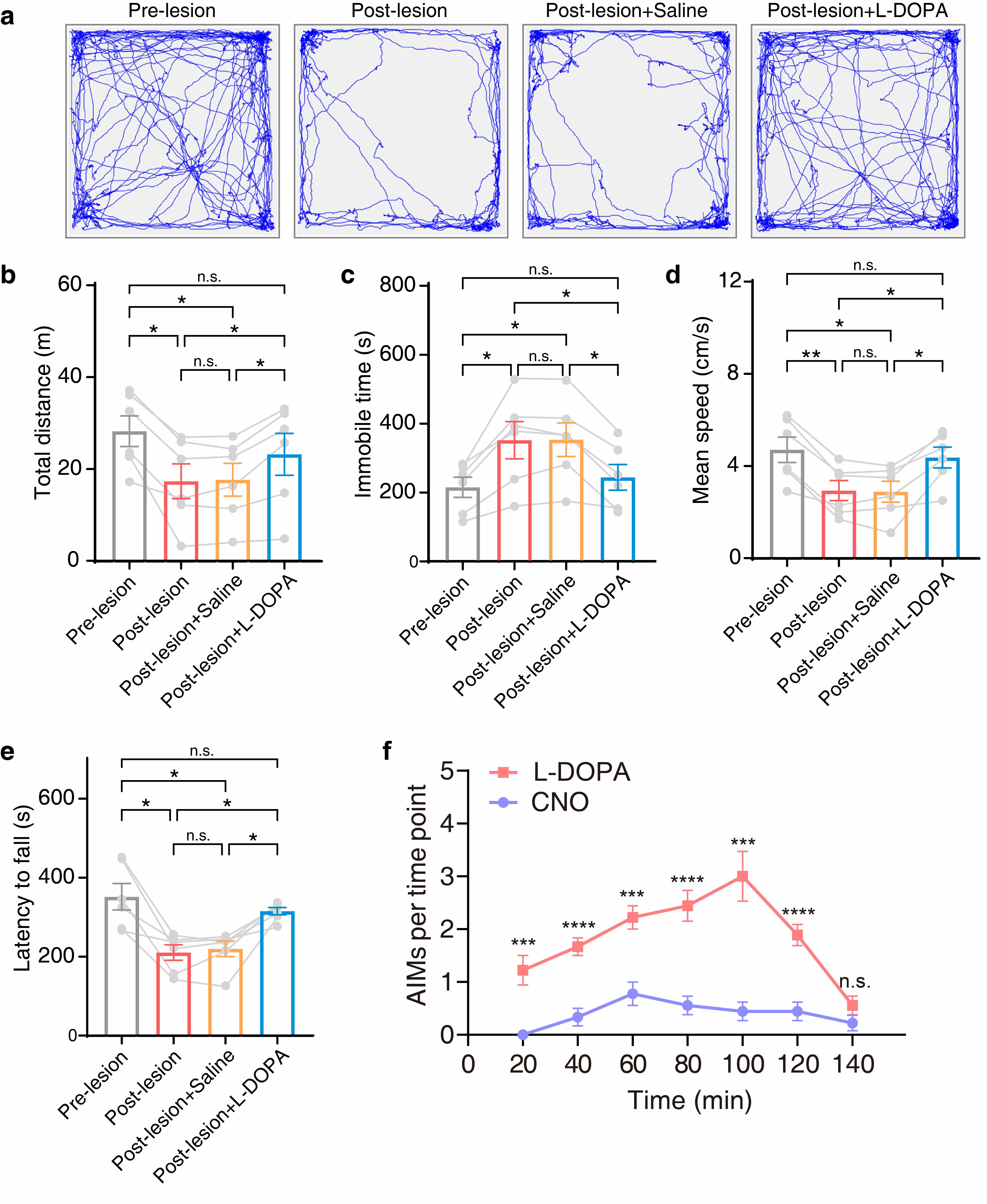


**Supplementary Figure 7.** Chemogenetic activation of D1-MSNs barely induced dyskinesia-like behavior in mice.

**a**, Representative locomotor trajectories during open-field tests before and after PD modeling, and after saline or L-DOPA treatment in lesioned mice.

**b-e**, Quantification of total distance traveled (**b**), time spent immobile (**c**), and mean speed (**d**) in an open-field test and latency to fall off the rotating rod (**e**); n=6 mice per group, data are represented as mean ± SEM, one-way ANOVA with Tukey’s post-hoc test, **p* < 0.05, ***p* < 0.01, n.s., not significant.

**f**, Time courses of AIM scores during the 140-minute test sessions (scored every 20 minutes until 140 minutes); n=7 mice per group, data are represented as mean ± SEM, one-way ANOVA with Tukey’s post-hoc test, ****p* < 0.001, *****p* < 0.0001, n.s., not significant.


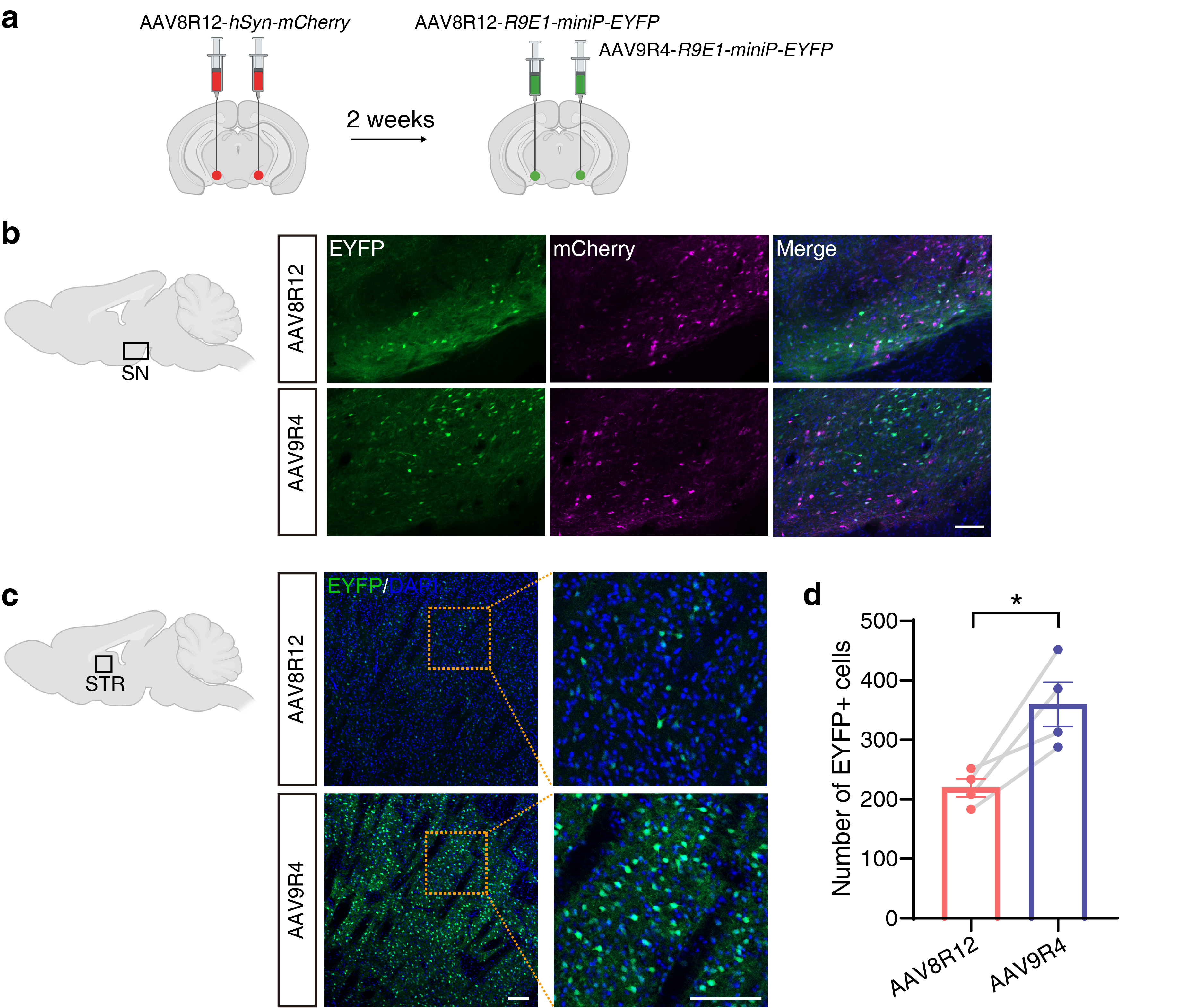


**Supplementary Figure 8.** AAV9R4 efficiently transduced striatal neurons in mice previously exposed to AAV8R12.

**a,b,** Schematic (**a**) and representative images (**b**) showing bilateral SNr injections of AAV8R12-*hSyn-mCherry* (magenta), followed by injections of AAV9R4-*R9E1-miniP-EYFP* (green) in right SNr, and AAV8R12-*R9E1-miniP-EYFP* (green) in left SNr, respectively. Scale bar, 100 μm.

**c,d,** Representative images (**c**) and quantifications (**d**) of labeled striatal MSNs (green) by AAV9R4-*R9E1-miniP-EYFP* or AAV8R12-*R9E1-miniP-EYFP* in mice previously exposed to AAV8R12. Scale bars, 100 μm. n=4 mice per group, data are represented as mean ± SEM, two-tailed paired *t*-test, **p* < 0.05.

| **Variants** | **Sequences** |
| --- | --- |
| AAV9R1 | atggctgccgatggttatcttccagattggctcgaggacaaccttagtgaaggaattcgcgagtggtgggctttgaaacctggagcccctcaacccaaggcaaatcaacaacatcaagacaacgctcgaggtcttgtgcttccgggttacaaataccttggacccggcaacggactcgacaagggggagccggtcaacgcagcagacgcggcggccctcgagcacgacaaggcctacgaccagcagctcaaggccggagacaacccgtacctcaagtacaaccacgccgacgccgagttccaggagcggctcaaagaagatacgtcttttgggggcaacctcgggcgagcagtcttccaggccaaaaagaggcttcttgaacctcttggtctggttgaggaagcggctaagacggctcctggaaagaagaggcctgtagagcagtctcctcaggaaccggactcctccgcgggtattggcaaatcgggtgcacagcccgctaaaaagagactcaatttcggtcagactggcgacacagagtcagtcccagaccctcaaccaatcggagaacctcccgcagccccctcaggtgtgggatctcttacaatggcttcaggtggtggcgcaccagtggcagacaataacgaaggtgccgatggagtgggtagttcctcgggaaattggcattgcgattcccaatggctgggggacagagtcatcaccaccagcacccgaacctgggccctgcccacctacaacaatcacctctacaagcaaatctccaacagcacatctggaggatcttcaaatgacaacgcctacttcggctacagcaccccctgggggtattttgacttcaacagattccactgccacttctcaccacgtgactggcagcgactcatcaacaacaactggggattccggcctaagcgactcaacttcaagctcttcaacattcaggtcaaagaggttacggacaacaatggagtcaagaccatcgccaataaccttaccagcacggtccaggtcttcacggactcagactatcagctcccgtacgtgctcgggtcggctcacgagggctgcctcccgccgttcccagcggacgttttcatgattcctcagtacgggtatctgacgcttaatgatggaagccaggccgtgggtcgttcgtccttttactgcctggaatatttcccgtcgcaaatgctaagaacgggtaacaacttccagttcagctacgagtttgagaacgtacctttccatagcagctacgctcacagccaaagcctggaccgactaatgaatccactcatcgaccaatacttgtactatctctcaaagactattaacggttctggacagaatcaacaaacgctaaaattcagtgtggccggacccagcaacatggctgtccagggaagaaactacatacctggacccagctaccgacaacaacgtgtctcaaccactgtgactcaaaacaacaacagcgaatttgcttggcctggagcttcttcttgggctctcaatggacgtaatagcttgatgaatcctggacctgctatggccagccacaaagaaggagaggaccgtttctttcctttgtctggatctttaatttttggcaaacaaggaactggaagagacaacgtggatgcggacaaagtcatgataaccaacgaagaagaaattaaaactactaacccggtagcaacggagtcctatggacaagtggccacaaaccaccagagcggaGATTCTTTGTCTTTTAATAAGgcacaggcgcagaccggctgggttcaaaaccaaggaatacttccgggtatggtttggcaggacagagatgtgtacctgcaaggacccatttgggccaaaattcctcacacggacggcaactttcacccttctccgctgatgggagggtttggaatgaagcacccgcctcctcagatcctcatcaaaaacacacctgtacctgcggatcctccaacggccttcaacaaggacaagctgaactctttcatcacccagtattctactggccaagtcagcgtggagatcgagtgggagctgcagaaggaaaacagcaagcgctggaacccggagatccagtacacttccaactattacaagtctaataatgttgaatttgctgttaatactgaaggtgtatatagtgaaccccgccccattggcaccagatacctgactcgtaatctgtaa |
| AAV9R2 | atggctgccgatggttatcttccagattggctcgaggacaaccttagtgaaggaattcgcgagtggtgggctttgaaacctggagcccctcaacccaaggcaaatcaacaacatcaagacaacgctcgaggtcttgtgcttccgggttacaaataccttggacccggcaacggactcgacaagggggagccggtcaacgcagcagacgcggcggccctcgagcacgacaaggcctacgaccagcagctcaaggccggagacaacccgtacctcaagtacaaccacgccgacgccgagttccaggagcggctcaaagaagatacgtcttttgggggcaacctcgggcgagcagtcttccaggccaaaaagaggcttcttgaacctcttggtctggttgaggaagcggctaagacggctcctggaaagaagaggcctgtagagcagtctcctcaggaaccggactcctccgcgggtattggcaaatcgggtgcacagcccgctaaaaagagactcaatttcggtcagactggcgacacagagtcagtcccagaccctcaaccaatcggagaacctcccgcagccccctcaggtgtgggatctcttacaatggcttcaggtggtggcgcaccagtggcagacaataacgaaggtgccgatggagtgggtagttcctcgggaaattggcattgcgattcccaatggctgggggacagagtcatcaccaccagcacccgaacctgggccctgcccacctacaacaatcacctctacaagcaaatctccaacagcacatctggaggatcttcaaatgacaacgcctacttcggctacagcaccccctgggggtattttgacttcaacagattccactgccacttctcaccacgtgactggcagcgactcatcaacaacaactggggattccggcctaagcgactcaacttcaagctcttcaacattcaggtcaaagaggttacggacaacaatggagtcaagaccatcgccaataaccttaccagcacggtccaggtcttcacggactcagactatcagctcccgtacgtgctcgggtcggctcacgagggctgcctcccgccgttcccagcggacgttttcatgattcctcagtacgggtatctgacgcttaatgatggaagccaggccgtgggtcgttcgtccttttactgcctggaatatttcccgtcgcaaatgctaagaacgggtaacaacttccagttcagctacgagtttgagaacgtacctttccatagcagctacgctcacagccaaagcctggaccgactaatgaatccactcatcgaccaatacttgtactatctctcaaagactattaacggttctggacagaatcaacaaacgctaaaattcagtgtggccggacccagcaacatggctgtccagggaagaaactacatacctggacccagctaccgacaacaacgtgtctcaaccactgtgactcaaaacaacaacagcgaatttgcttggcctggagcttcttcttgggctctcaatggacgtaatagcttgatgaatcctggacctgctatggccagccacaaagaaggagaggaccgtttctttcctttgtctggatctttaatttttggcaaacaaggaactggaagagacaacgtggatgcggacaaagtcatgataaccaacgaagaagaaattaaaactactaacccggtagcaacggagtcctatggacaagtggccacaaaccaccagagcggaGGTGTGGAGCCTCGTGCGGGTgcacaggcgcagaccggctgggttcaaaaccaaggaatacttccgggtatggtttggcaggacagagatgtgtacctgcaaggacccatttgggccaaaattcctcacacggacggcaactttcacccttctccgctgatgggagggtttggaatgaagcacccgcctcctcagatcctcatcaaaaacacacctgtacctgcggatcctccaacggccttcaacaaggacaagctgaactctttcatcacccagtattctactggccaagtcagcgtggagatcgagtgggagctgcagaaggaaaacagcaagcgctggaacccggagatccagtacacttccaactattacaagtctaataatgttgaatttgctgttaatactgaaggtgtatatagtgaaccccgccccattggcaccagatacctgactcgtaatctgtaa |
| AAV9R3 | atggctgccgatggttatcttccagattggctcgaggacaaccttagtgaaggaattcgcgagtggtgggctttgaaacctggagcccctcaacccaaggcaaatcaacaacatcaagacaacgctcgaggtcttgtgcttccgggttacaaataccttggacccggcaacggactcgacaagggggagccggtcaacgcagcagacgcggcggccctcgagcacgacaaggcctacgaccagcagctcaaggccggagacaacccgtacctcaagtacaaccacgccgacgccgagttccaggagcggctcaaagaagatacgtcttttgggggcaacctcgggcgagcagtcttccaggccaaaaagaggcttcttgaacctcttggtctggttgaggaagcggctaagacggctcctggaaagaagaggcctgtagagcagtctcctcaggaaccggactcctccgcgggtattggcaaatcgggtgcacagcccgctaaaaagagactcaatttcggtcagactggcgacacagagtcagtcccagaccctcaaccaatcggagaacctcccgcagccccctcaggtgtgggatctcttacaatggcttcaggtggtggcgcaccagtggcagacaataacgaaggtgccgatggagtgggtagttcctcgggaaattggcattgcgattcccaatggctgggggacagagtcatcaccaccagcacccgaacctgggccctgcccacctacaacaatcacctctacaagcaaatctccaacagcacatctggaggatcttcaaatgacaacgcctacttcggctacagcaccccctgggggtattttgacttcaacagattccactgccacttctcaccacgtgactggcagcgactcatcaacaacaactggggattccggcctaagcgactcaacttcaagctcttcaacattcaggtcaaagaggttacggacaacaatggagtcaagaccatcgccaataaccttaccagcacggtccaggtcttcacggactcagactatcagctcccgtacgtgctcgggtcggctcacgagggctgcctcccgccgttcccagcggacgttttcatgattcctcagtacgggtatctgacgcttaatgatggaagccaggccgtgggtcgttcgtccttttactgcctggaatatttcccgtcgcaaatgctaagaacgggtaacaacttccagttcagctacgagtttgagaacgtacctttccatagcagctacgctcacagccaaagcctggaccgactaatgaatccactcatcgaccaatacttgtactatctctcaaagactattaacggttctggacagaatcaacaaacgctaaaattcagtgtggccggacccagcaacatggctgtccagggaagaaactacatacctggacccagctaccgacaacaacgtgtctcaaccactgtgactcaaaacaacaacagcgaatttgcttggcctggagcttcttcttgggctctcaatggacgtaatagcttgatgaatcctggacctgctatggccagccacaaagaaggagaggaccgtttctttcctttgtctggatctttaatttttggcaaacaaggaactggaagagacaacgtggatgcggacaaagtcatgataaccaacgaagaagaaattaaaactactaacccggtagcaacggagtcctatggacaagtggccacaaaccaccagagcggaCGTCCGACGCGTGATTCTACTgcacaggcgcagaccggctgggttcaaaaccaaggaatacttccgggtatggtttggcaggacagagatgtgtacctgcaaggacccatttgggccaaaattcctcacacggacggcaactttcacccttctccgctgatgggagggtttggaatgaagcacccgcctcctcagatcctcatcaaaaacacacctgtacctgcggatcctccaacggccttcaacaaggacaagctgaactctttcatcacccagtattctactggccaagtcagcgtggagatcgagtgggagctgcagaaggaaaacagcaagcgctggaacccggagatccagtacacttccaactattacaagtctaataatgttgaatttgctgttaatactgaaggtgtatatagtgaaccccgccccattggcaccagatacctgactcgtaatctgtaa |
| AAV9R4 | atggctgccgatggttatcttccagattggctcgaggacaaccttagtgaaggaattcgcgagtggtgggctttgaaacctggagcccctcaacccaaggcaaatcaacaacatcaagacaacgctcgaggtcttgtgcttccgggttacaaataccttggacccggcaacggactcgacaagggggagccggtcaacgcagcagacgcggcggccctcgagcacgacaaggcctacgaccagcagctcaaggccggagacaacccgtacctcaagtacaaccacgccgacgccgagttccaggagcggctcaaagaagatacgtcttttgggggcaacctcgggcgagcagtcttccaggccaaaaagaggcttcttgaacctcttggtctggttgaggaagcggctaagacggctcctggaaagaagaggcctgtagagcagtctcctcaggaaccggactcctccgcgggtattggcaaatcgggtgcacagcccgctaaaaagagactcaatttcggtcagactggcgacacagagtcagtcccagaccctcaaccaatcggagaacctcccgcagccccctcaggtgtgggatctcttacaatggcttcaggtggtggcgcaccagtggcagacaataacgaaggtgccgatggagtgggtagttcctcgggaaattggcattgcgattcccaatggctgggggacagagtcatcaccaccagcacccgaacctgggccctgcccacctacaacaatcacctctacaagcaaatctccaacagcacatctggaggatcttcaaatgacaacgcctacttcggctacagcaccccctgggggtattttgacttcaacagattccactgccacttctcaccacgtgactggcagcgactcatcaacaacaactggggattccggcctaagcgactcaacttcaagctcttcaacattcaggtcaaagaggttacggacaacaatggagtcaagaccatcgccaataaccttaccagcacggtccaggtcttcacggactcagactatcagctcccgtacgtgctcgggtcggctcacgagggctgcctcccgccgttcccagcggacgttttcatgattcctcagtacgggtatctgacgcttaatgatggaagccaggccgtgggtcgttcgtccttttactgcctggaatatttcccgtcgcaaatgctaagaacgggtaacaacttccagttcagctacgagtttgagaacgtacctttccatagcagctacgctcacagccaaagcctggaccgactaatgaatccactcatcgaccaatacttgtactatctctcaaagactattaacggttctggacagaatcaacaaacgctaaaattcagtgtggccggacccagcaacatggctgtccagggaagaaactacatacctggacccagctaccgacaacaacgtgtctcaaccactgtgactcaaaacaacaacagcgaatttgcttggcctggagcttcttcttgggctctcaatggacgtaatagcttgatgaatcctggacctgctatggccagccacaaagaaggagaggaccgtttctttcctttgtctggatctttaatttttggcaaacaaggaactggaagagacaacgtggatgcggacaaagtcatgataaccaacgaagaagaaattaaaactactaacccggtagcaacggagtcctatggacaagtggccacaaaccaccagagcggaGTGTTGTCTTCTGTTGGGGATgcacaggcgcagaccggctgggttcaaaaccaaggaatacttccgggtatggtttggcaggacagagatgtgtacctgcaaggacccatttgggccaaaattcctcacacggacggcaactttcacccttctccgctgatgggagggtttggaatgaagcacccgcctcctcagatcctcatcaaaaacacacctgtacctgcggatcctccaacggccttcaacaaggacaagctgaactctttcatcacccagtattctactggccaagtcagcgtggagatcgagtgggagctgcagaaggaaaacagcaagcgctggaacccggagatccagtacacttccaactattacaagtctaataatgttgaatttgctgttaatactgaaggtgtatatagtgaaccccgccccattggcaccagatacctgactcgtaatctgtaa |
| AAV9R5 | atggctgccgatggttatcttccagattggctcgaggacaaccttagtgaaggaattcgcgagtggtgggctttgaaacctggagcccctcaacccaaggcaaatcaacaacatcaagacaacgctcgaggtcttgtgcttccgggttacaaataccttggacccggcaacggactcgacaagggggagccggtcaacgcagcagacgcggcggccctcgagcacgacaaggcctacgaccagcagctcaaggccggagacaacccgtacctcaagtacaaccacgccgacgccgagttccaggagcggctcaaagaagatacgtcttttgggggcaacctcgggcgagcagtcttccaggccaaaaagaggcttcttgaacctcttggtctggttgaggaagcggctaagacggctcctggaaagaagaggcctgtagagcagtctcctcaggaaccggactcctccgcgggtattggcaaatcgggtgcacagcccgctaaaaagagactcaatttcggtcagactggcgacacagagtcagtcccagaccctcaaccaatcggagaacctcccgcagccccctcaggtgtgggatctcttacaatggcttcaggtggtggcgcaccagtggcagacaataacgaaggtgccgatggagtgggtagttcctcgggaaattggcattgcgattcccaatggctgggggacagagtcatcaccaccagcacccgaacctgggccctgcccacctacaacaatcacctctacaagcaaatctccaacagcacatctggaggatcttcaaatgacaacgcctacttcggctacagcaccccctgggggtattttgacttcaacagattccactgccacttctcaccacgtgactggcagcgactcatcaacaacaactggggattccggcctaagcgactcaacttcaagctcttcaacattcaggtcaaagaggttacggacaacaatggagtcaagaccatcgccaataaccttaccagcacggtccaggtcttcacggactcagactatcagctcccgtacgtgctcgggtcggctcacgagggctgcctcccgccgttcccagcggacgttttcatgattcctcagtacgggtatctgacgcttaatgatggaagccaggccgtgggtcgttcgtccttttactgcctggaatatttcccgtcgcaaatgctaagaacgggtaacaacttccagttcagctacgagtttgagaacgtacctttccatagcagctacgctcacagccaaagcctggaccgactaatgaatccactcatcgaccaatacttgtactatctctcaaagactattaacggttctggacagaatcaacaaacgctaaaattcagtgtggccggacccagcaacatggctgtccagggaagaaactacatacctggacccagctaccgacaacaacgtgtctcaaccactgtgactcaaaacaacaacagcgaatttgcttggcctggagcttcttcttgggctctcaatggacgtaatagcttgatgaatcctggacctgctatggccagccacaaagaaggagaggaccgtttctttcctttgtctggatctttaatttttggcaaacaaggaactggaagagacaacgtggatgcggacaaagtcatgataaccaacgaagaagaaattaaaactactaacccggtagcaacggagtcctatggacaagtggccacaaaccaccagagcggaTCTGCGTTGACGGCGCAGCTTgcacaggcgcagaccggctgggttcaaaaccaaggaatacttccgggtatggtttggcaggacagagatgtgtacctgcaaggacccatttgggccaaaattcctcacacggacggcaactttcacccttctccgctgatgggagggtttggaatgaagcacccgcctcctcagatcctcatcaaaaacacacctgtacctgcggatcctccaacggccttcaacaaggacaagctgaactctttcatcacccagtattctactggccaagtcagcgtggagatcgagtgggagctgcagaaggaaaacagcaagcgctggaacccggagatccagtacacttccaactattacaagtctaataatgttgaatttgctgttaatactgaaggtgtatatagtgaaccccgccccattggcaccagatacctgactcgtaatctgtaa |

**Table S1.** The DNA sequences of AAV9 capsid variants (VP1)

Sequences encoding 7-mer peptides are in uppercase and underlined.

| Capsids | pTM scores |
| --- | --- |
| AAV9 | 0.94 |
| AAV9R1 | 0.93 |
| AAV9R2 | 0.94 |
| AAV9R3 | 0.93 |
| AAV9R4 | 0.93 |
| AAV9R5 | 0.94 |

**Table S2.** The predicted template modeling (pTM) score of VP3 proteins by AlphaFold3

| AAV capsid | Genetic payload | Titer (VG/mL) |
| --- | --- | --- |
| AAV9 | hSyn-mCherry-WPRE-BGHpA | 3.74 × 10^13^ |
| AAV9R1 | hSyn-mCherry-WPRE-BGHpA | 3.20 × 10^13^ |
| AAV9R2 | hSyn-mCherry-WPRE-BGHpA | 1.53 × 10^13^ |
| AAV9R3 | hSyn-mCherry-WPRE-BGHpA | 1.14 × 10^13^ |
| AAV9R4 | hSyn-mCherry-WPRE-BGHpA | 1.22 × 10^13^ |
| AAV9R5 | hSyn-mCherry-WPRE-BGHpA | 2.38 × 10^13^ |

**Table S3.** AAV vector production

Single-stranded AAV2 ITRs were used to flank genetic payload.

| **Enhancer** | **Genomic Coordinate/5’ end** | **Length (bp)** | **Sequence** |
| --- | --- | --- | --- |
| *R9E1* | GRCh38:17:65144153(+) | 503 | CCAGGCTTATTAGCAGGAGTACATCCTCCTCTGTTAGAAAAACAACTCTGCGGTTCTGGGTCACTTTTCCCATCTTTGTATAGAAAAGTACCGATTATTATTGTTCCCCCGGGTGCCTTCTCCAGCTTAAAGGAAAATTCCAGCCCCAGAGATCTTCCGCAGTGAGTGACAGCTCCTTAATGTGAGGAGCAGCGTGGGCGTGGTGGGTGGGATGTCAGAGCCATCAAAGCCCTCATTTCTGATCCCTCTGTGATGTCGGGGTTCCCTGGCACGCCCACTGTCATGCCTCAGCACATTGGCATCTGAGAGAGGAAAGGTGCAGCTGACACCCTGACCCCCTTCTCCCCGACACGGGGTCAGACAGGGTCCCGCGGTGGCCTGGTTGTGCAGCACTGACATGTTGCCTGGGAACGAAGCGGGGCTGATGCTTTGTCTTGGGAGGAGCTGGGAAGCAGAGTCTGTTTGGGAGTGAGAGTGTTGGCCTTTGAGGGTGAGCATGCCCC |
| *R9E2* | GRCh38:17:65145788(+) | 401 | CAGTTCAGCCCATAGAGCTAGAGTTCTGTGGTTTCCTGGGGAGACTGAGGAGACGCCTCAGAGGAATTTGTACGGGGAAAGGTCTGAGGAAAAAGGATCGGATCCCAATGGGCCGAACTCCCTCAAAATGTTCCCGAGGTCCACGTGACGTTCCCTCCTCCCACCCAGCCCTTCTTTCTGTGAAGGGTGACACACCTCCTAGAAAGAAATCTGACTCCCCTCATTGTGTTTTTAAAATAACTCTCATTATTCCTCCTCTGAAGGTTATTTTCAAACTCTTTATAACTCGATTTTTTTTTAAATGCAAAGACAAGGAATCAAATCTGCCAACTTTCTATCAGAGAAACTCGGGTTTCTAACTTTTAAAGTGGGATTATATGTCTTCTCCAATATATGGGCTC |
| *R9E3* | GRCh38:17:65156462(+) | 498 | CCAGCTGCTGTAGAAACAACACAGGGAGAAGCCCTGGTCCTGCAACAACAGCACCGATAAAAAAAAATTATTTAACTTCTTGGACGGCAGGCGTCCCAGGGCTTCCTCGGGACAAAGGTGGCTGATGCGGGAGGCTGAGCAGCCTGCCAGGCTGTCAACGTAGACCGCGTGGTACAGATGCTGGTGGCACCATAATCTCATTTGATCTATTTCGGGAGGCAGTCAAGTGGATGAGAAAGCCCTCGGCTCCTCGGTGGAGAGAGCTAATCAGTCTGATGTCTGCCTAGGTCTGTCTCTCTGCCTGCGCACCACTGTGTAGCATTTAAAGAGAGAGACTTGGTGTGGAAAGTCTAAACACCCAGTCCTAGGAGTTTAGATGAAGGATTTTGCTTTTGAACAGCCATCCAGGAGTGAATGATGCTAAGAGCAACAGAGGGAGAACCATGGAAAGCCATGACAGTGTGCATGTGGACACTGACAGAGATCATGGCTGGACAT |
| *R9E4* | GRCh38:17:65166689(+) | 474 | GGACCCGTGGGGTAAATCAGAGAGTGACTCTATTTACCAGGCTCTCCAGCATTGTGGAAACTGCAATGCCTCAGGCTGGTTGTGGCCCATGCTCCTGAGGTCAGGGGCTAGTTAATGTGGTAGAAAATCCAGTTAGGCTGTCAGGGGGAAGTTTGAAAACAATGTTTATATTTAATTTCCAGAGGAGGCTGACGGAACGCCCAAATGGAATGGCGCTGTTCACCCATCTGGCTCCCTGAGTGTTATGATGTTTTTCACAGTACGTTAACGGGGAGATGAATTCGCCGACTCTGTCTTGCAGAGGGCGTGTGCGTCATCCCACGTTGCCTGGGAAAACAAGCATTAACAAGTGTGAGCGCGGGTCTCCGTGGAAATGGTACAGAAGGGGGCCCACGGCGGGATCATTAGTGTTACTTTGCCCCTGGAGGAAGAAGGCCCTGCGTCATTTCCCATCCAGAGTGGGAAAGGGAGAGA |

**Table S4.** Candidate enhancers

This table shows the names, genomic positions, sizes, and sequences of candidate enhancers.
